# Estimating protein isoform abundances with PAQu

**DOI:** 10.64898/2026.04.20.719668

**Published:** 2026-04-22

**Authors:** Lorenzo Testa, Lambertus Klei, Alesia Rengle, Anastasia Yocum, David A. Lewis, Bernie Devlin, Kathryn Roeder, Matthew L. MacDonald

**Affiliations:** 1 Department of Statistics and Data Science, Carnegie Mellon University, Pittsburgh, PA 15213; 2 L’EMbeDS, Sant’Anna School of Advanced Studies, Pisa, IT 56127; 3 Department of Psychiatry, University of Pittsburgh School of Medicine, Pittsburgh, PA 15213; 4 Department of Statistics, University of Pittsburgh, Pittsburgh, PA 15260; 5 A2IDEA, 674 S. Wagner Rd., Ann Arbor, MI 48103; 6 Center for Neuroscience, University of Pittsburgh, Pittsburgh, PA, USA 15213; 7 Department of Computational Biology, Carnegie Mellon University, Pittsburgh, PA 15213; 8 Biomedical Mass Spectrometry Center, University of Pittsburgh, Pittsburgh, PA 15260

**Keywords:** Transcript, Isoform, Bayesian estimation, schizophrenia, trans-synaptic signaling

## Abstract

A single gene can encode multiple versions of a protein, dubbed isoforms, with varying functionality. Cellular control of isoform abundances is critical for multiple aspects of biology and is only partially regulated by transcript levels. While long-read sequencing facilitates transcript quantification, quantifying the resulting protein isoforms on a large scale is a major challenge, complicating biological interpretation of transcript alterations. Standard “bottom up” mass spectrometry can assess only short portions of isoforms called peptides, and these peptides often map onto more than one isoform. We introduce PAQu, a novel Bayesian method that leverages multiomic information from the peptidome and transcriptome to provide accurate estimates of isoform abundance even when peptide mapping is ambiguous. PAQu offers several advantages over existing methods in a unified framework. It provides uncertainty quantification, integrates multiomic information for improved accuracy, and provides a rigorous framework for hypothesis testing. Extensive simulations show that PAQu consistently outperforms competing methods in detecting differentially expressed protein isoforms and estimating their abundances. We use PAQu to investigate differences in isoform abundance levels between people with schizophrenia and control subjects, confirming a long held hypothesis that levels of the C4A isoform of Complement Component 4 are increased in schizophrenia while C4B is not. These results demonstrate that PAQu can identify significant variations in isoform abundance levels not previously possible.

## Introduction

Alternative splicing is a crucial post-transcriptional process (1, 2) that generates multiple protein isoforms from a single gene, thereby contributing to the complexity of eukaryotic organisms. Dysregulation of this process has been linked to various diseases, including cancer (3, 4), neurodegenerative and psychiatric disorders (5–7), and developmental abnormalities (8–10). Despite the significant role of alternative splicing in cellular function (2), accurate and comprehensive quantification of protein isoforms remains a major challenge in proteomics (11, 12).

In a typical shotgun proteomics experiment, proteins are first digested into peptides and then analyzed by liquid chromatography tandem mass spectrometry (LC-MS/MS). The resulting spectra are used to identify and quantify peptides in digested samples. Although this approach is effective for analyzing individual peptides, it often falls short when the goal is to characterize protein isoforms (13). In particular, a significant challenge in protein isoform identification and quantification is the presence of *shared* peptides (11–13), which can map onto multiple isoforms of the same protein or even to different proteins due to sequence similarity. Additionally, peptides unique to individual isoforms are typically less abundant compared to those that are shared.

To overcome this challenge, standard practices either collapse proteins with shared peptides into protein groups or ignore shared peptides altogether, which limit isoform characterization (14–16). Several other strategies aggregate peptide abundances into protein isoform estimates by taking summary statistics (e.g., average, sum, maximum) without considering whether peptides are shared or isoform-specific. These approaches are reflected in the default settings in the commonly used informatics programs, such as Proteome Discoverer (17) and MS Fragger (18). As a consequence, standard methods often struggle to distinguish between isoforms with high sequence similarity, leading to underestimation of isoform diversity or potential misinterpretation of biological processes.

To improve isoform quantification, recent approaches have attempted to integrate transcriptomic data with proteomic data, using the relationship between gene expression and protein production. These methods offer a promising direction for tackling the challenge of protein quantification. In particular, several studies such (19–21) have introduced techniques that use transcript expression levels as a prior source of information to estimate isoform abundances. However, despite their advances, these methods come with several limitations. For example, Carlyle et al. (19) employ an expectation-maximization (EM) algorithm that does not provide any direct measure of uncertainty quantification. Miller et al. (20) rely on long-read RNA-seq, an expensive technique to obtain transcript data. Bollon et al. (21), exploiting the conjugacy between Dirichlet and Multinomial distributions to accelerate computation, need to discretize the data, resulting in reduced precision. Moreover, none of these methods provides a direct way to evaluate the presence of differentially abundant protein isoforms between different groups of samples.

To overcome these shortcomings, we introduce a new method – which we call PAQu – for accurate Protein isoform Abundance Quantification. PAQu combines the strengths of transcriptomic and proteomic data, exploiting complementary insights from RNA-Seq and mass spectrometry. It offers robust uncertainty quantification for all estimated parameters, integrates external covariate information, elucidates the relationship between the transcriptome and the proteome, and enables simultaneous differential analysis, providing a comprehensive solution for isoform quantification. Complete PAQu code is available on GitHub at: https://github.com/testalorenzo/PAQu.

## Results

### Overview of PAQu

**Figure 1:**
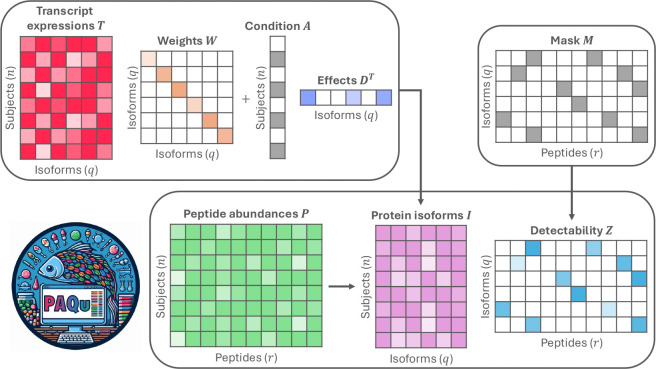
Summary of PAQu model. The model takes as input a transcript expression matrix T, a condition binary vector A, a detectability mask matrix M, and a peptide abundance matrix P. The output includes the effects of transcripts on protein isoforms W, the difference in protein isoform abundances across conditions D, and the estimated associations between isoforms and peptides Z.

PAQu is a Bayesian supervised factor analysis method designed for Protein isoform Abundance Quantification. It models protein isoform abundances as latent factors and estimates them by learning two key mappings: one from transcript expression levels to protein isoform abundances, and another from protein isoform to peptide abundances. Simultaneously, PAQu assesses the impact of binary conditions on isoform abundances, enabling analysis of differential protein abundance. Our method takes four inputs: a transcript expression matrix from tissue samples from n subjects, a peptide abundance matrix from tissue from the same (or possibly different) subjects, a detectability mask matrix indicating whether a peptide k can come from a protein isoform j, and a binary vector representing a condition for subjects, such as diagnosis (Fig. 1). PAQu assumes that transcript expression levels are proportional to the corresponding protein isoform abundances, and that peptide abundances are related to their respective protein isoforms. This naturally leads to a two-layer model. In the first layer, the transcript expression matrix T is transformed into the latent protein isoform matrix I through a diagonal weight matrix W.

In the second layer, the peptide abundance matrix P is modeled as the product of the protein isoform matrix I and the detectability matrix Z (whose entries can be zero according to the detectability mask matrix provided as input). Moreover, for differential protein abundance analysis, the first layer also incorporates the relationship between the binary condition vector A and the transcript expression matrix T through the vector of effects D. Conceptually, the first layer performs multivariate linear regression of protein isoforms I on transcripts T and condition A, while the second layer applies factor analysis to P. By constraining, in the first layer, the direction of the protein isoform factors within the space spanned by the transcripts, PAQu supervises, in the second layer, the estimation of the protein factors. Finally, if available, PAQu can also take advantage of the information coming from external covariates, easily inserting them into the previous model (see Methods).

Although this two-layer problem is not identifiable in the traditional frequentist sense, imposing prior distributions on the unknowns allows estimation of the quantities of interest. Specifically, for the PAQu model, we use Gaussian priors for the protein isoform abundances I, the weights in W, the effects in D, and truncated Gaussian priors for the detectability factors in Z. For situations with a high signal-to-noise ratio, users can opt for a sparser “spike-and-slab” prior (22), which offers more conservative inference by selectively shrinking smaller effects.

We implement a Gibbs sampling algorithm to generate posterior samples of the model parameters (23). The significance of any effect can be measured by the local false sign rate (LFSR) metric (24), a summary of the posterior distribution akin to local false discovery rate (see Methods). Similarly, for parameters with sparse priors, posterior inclusion probability (PIP) can be easily computed.

PAQu generates several outputs: estimated associations between transcripts and protein isoforms, inferred associations between protein isoforms and peptides, estimated abundances of protein isoforms, and a list of differentially abundant protein isoforms, which can be interpreted, for instance, through gene ontology (GO) enrichment analysis of genes associated with each isoform.

All of these estimated quantities are accompanied by a measure of their uncertainty.

### Simulation results provide strong motivation for PAQu

We evaluate the performance of PAQu in two main simulation settings. In the first setting, which we refer to as “easy”, we generate data without shared peptides. Specifically, we sample one transcript (q=1) from a Normal distribution, map it into a protein isoform with random conversion weight, add an effect due to a binary condition, and then split it into two peptides (r=2) using randomly assigned detectability scores (see Methods). This setting serves as a reference point to compare PAQu with conventional methods for protein quantification. We run each simulation experiment with three different sample sizes (n=100,200,500), three effect sizes for the binary condition $\left(D_{j}=0.33,0.66,1\right)$, and with 25 different seeds. In the second, more challenging scenario – referred to as the “difficult” setting – we generate data that include shared peptides. Here, we sample five transcripts (q=5) from a multivariate Normal distribution, map them to their corresponding protein isoforms with random conversion weights, and introduce a binary condition effect to some isoforms. These isoforms are split into 10 peptides (r=10) using random detectability scores, with 30% of the detectability matrix entries being nonzero. Each simulation is run across three sample sizes (n=100,200,500), three effect sizes $\left(D_{j}=0.33,0.66,1\right)$, three numbers of differentially abundant isoforms $\left(\left|D^{act}\right|=1,2,3\right)$ and 25 different random seeds.

We exploit simulated data to assess the performance of various prior distributions in differential isoform abundance tests. In our simulations, the effects inferred by using the “spike-and-slab” prior are overly conservative, particularly when the signal or the sample size is small. In contrast, the Normal prior exhibits much less bias (SI Appendix, Fig. 6 and 7), which supports our decision to adopt the Normal prior as default. We then evaluate the ability of PAQu to recover and quantify differentially abundant protein isoforms. PAQu accurately estimates these effects, showing a slightly conservative behavior in the simulation setting “hard” in terms of recovery of differentially abundant isoforms at LFSR≤0.05 when the sample size and the signal are small (SI Appendix, Fig. 8 and 9). This leads to a modest increase in false negatives, as expected due to the conservative prior used and the extreme weakness of the signal.

**Figure 2:**
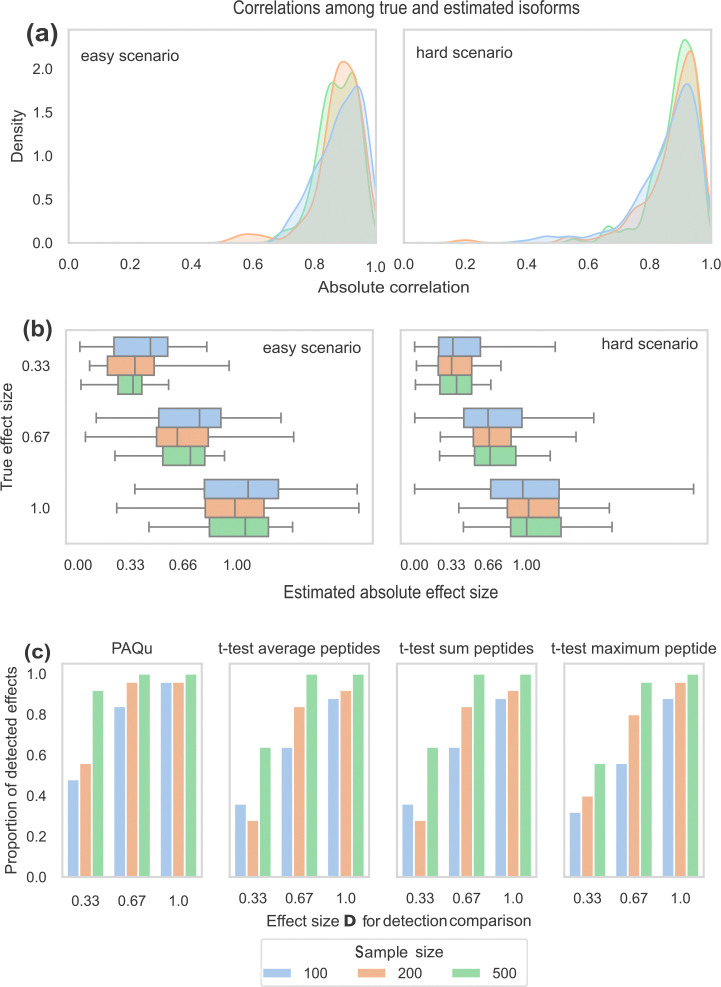
Simulation results. **(a)** Distributions of the absolute correlation values between true protein isoforms and the estimates inferred by PAQu under the “easy” (left) and “difficult” (right) scenarios. Different colors represent different sample sizes, ranging from 100 to 500. **(b)** Box plots of absolute effect sizes estimated by PAQu under the “easy” (left) and “difficult” (right) scenario. Different colors represent different sample sizes. For each box, the center line represents the median; the lower and upper hinges correspond to the first and third quartiles; the upper and lower whiskers span 2 times the interquartile range. **(c)** Comparison between the proportion of detected effects $D_{j}$ under the “easy” scenario between PAQu (LFSR ≤ 0.05), t-test on the average of peptide abundances, t-test on the sum of peptide abundances, t-test on the maximum peptide abundance (p-value ≤ 0.05). All simulations are repeated 25 times.

To assess the performance of PAQu in inferring the latent protein isoform factors, we measure the absolute correlation between the inferred and true isoform abundances. In all scenarios, the estimated protein factors show a strong correlation with the true factors (Fig. 2). As expected, the quality of the estimates increases with the sample size; at the same time, estimates tend to slightly worsen with increasing number of parameters to estimate.

We then compare the performance of PAQu in detecting differentially abundant isoforms with other commonly used tools: t-test on the average abundances of peptides, t-test on the sum of peptides, and t-test on the maximum peptide abundance. PAQu outperforms all these methods in all simulation settings (Fig. 2, SI Appendix, 8 and 9). These results demonstrate not only the performance gains PAQu can provide, but also the value of integrating data from both the transcriptome and the peptidome to achieve more precise isoform-level estimates.

Moreover, to further challenge PAQu and measure its performance under misspecification of the model, we evaluate simulations in which we assume that there is no link between transcript expression levels and protein isoform abundances (i.e. W=0). Remarkably, even when transcripts carry no information about protein isoforms, PAQu can still provide meaningful inference, significantly outperforming competitors (SI Appendix, Fig. 10, 11, and 12). To evaluate the contribution of information from transcript expressions, we performed simulations in which we compared the estimates of PAQu without transcripts, only average values between samples and full transcript expression levels. As the amount of transcript expression increases, so does the quality of isoform abundance estimates, measured by the absolute correlation between true and estimated abundances (SI Appendix, Fig. 13). Finally, we assess computational feasibility. Although T and P can be large, because Z is block diagonal, computations via the Gibbs sampler can proceed in parallel by block, making them efficient (SI Appendix, Fig. 14).

### PAQu reveals the effects of schizophrenia risk genes

We applied PAQu to the most deeply characterized proteomic dataset of human brain tissue for a psychiatric disorder. Quantitative proteomics using TMT labeling and deep offline fractionation was applied to postmortem dorsal anterior cingulate cortex gray matter from 56 individuals diagnosed with schizophrenia and 56 matched controls with no history of psychiatric or neurological conditions. While “protein" expression alterations have previously been observed in schizophrenia, here we report differentially abundant protein isoforms. Furthermore, we have complementary data on transcripts, based on RNA-seq measurements (25), in adjacent tissue sections from a subset of the same subjects. After collecting and preprocessing data (see Methods), we have protein and transcript data from n=103 subjects, 53 of whom are affected by schizophrenia, and consisting of q=9169 isoforms and r=79, 040 peptides. Groups of isoforms that shared exactly the same measured peptides, and therefore were not uniquely estimable, were rolled up into isoform groups, which resulted in 1440 isoform groups representing 3338 isoforms and 5831 single peptides. The isoform groups can be divided into 1396 and 44 groups mapping to a single or multiple genes, respectively. PAQu calculations were performed using the combined 7271 single and grouped isoforms.

We ran PAQu with 10 different MCMC chains, each consisting of 3000 iterations (the first 2000 are burn-in), both to measure PAQu consistency and to gauge the stability of our findings. For differential case-control abundance, the SI Appendix, Fig. 15 shows the detection frequency results across chains at LFSR ≤ 0.05. Out of 7271 isoforms, PAQu never selects 6211 isoforms (85.4%) as differentially abundant, and always detects 541 isoforms (7.4%). The remaining 519 (7.1%) isoforms are selected at least once, but only 179 of them are detected more than 5 times. The high degree of consistency among runs further strengthens confidence in the interpretation of the findings obtained by using PAQu.

**Figure 3:**
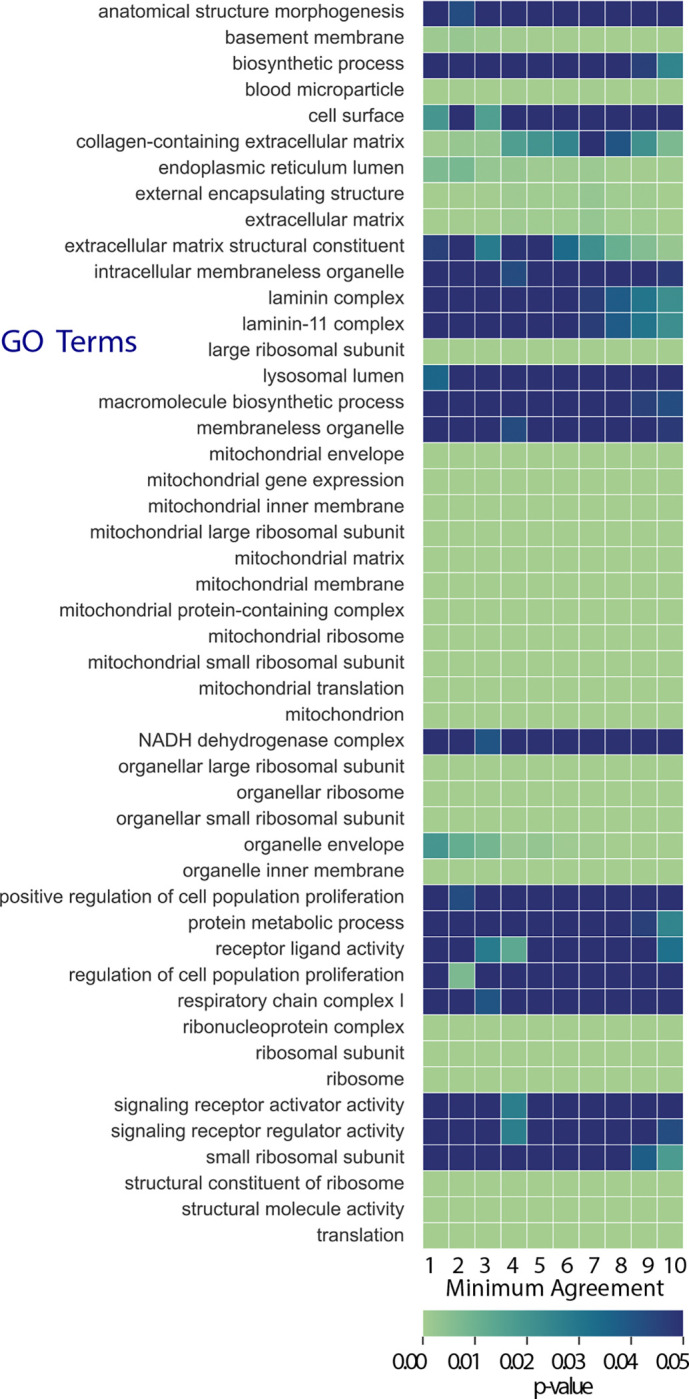
GO enrichment analysis. Isoforms are characterized by the number of times they are selected across PAQu MCMC chains. Then, for each level of minimum agreement factor γ among PAQu runs, we map protein isoforms meeting this criterion back to their associated genes and then perform Gene Ontology (GO) enrichment analysis.

We group isoforms on the basis of the number of times they are selected across MCMC chains. Then, for each level of minimum agreement factor γ among PAQu runs, we map protein isoforms back to their associated genes, and then perform Gene Ontology (GO) enrichment analysis (Fig. 3). Notably, for more lenient thresholds for significance, synaptic and neuronal biology emerge from the gene set, whereas themes involving energetics (mitochondrial) and protein synthesis (ribosomal) are always over-represented.

### PAQu sheds light on the relationship between transcriptome, proteome, and peptidome

**Figure 4:**
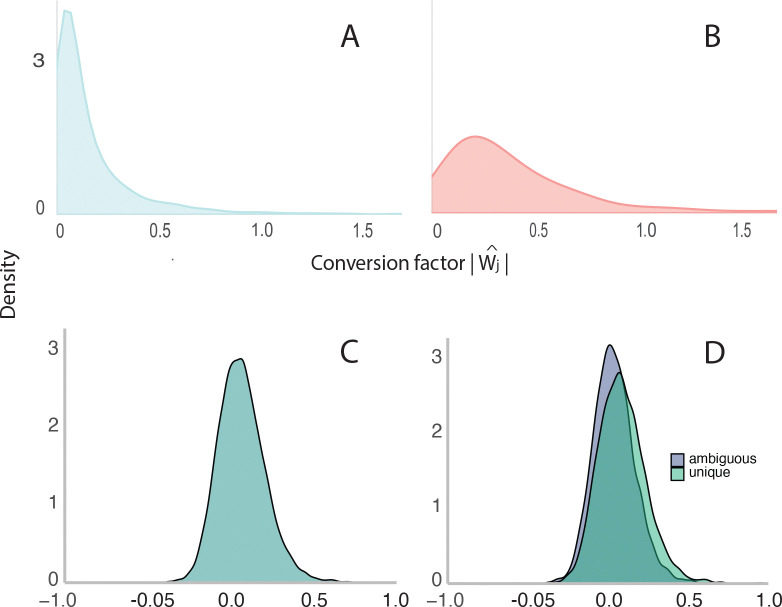
Relationship of transcripts and protein isoform abundance within individuals. (A-B) Transcripts and protein isoforms that show biological variation (γ>5): (A) for all, (B) detected by t-test on transcripts and PAQu. (C) Correlation between all transcripts and their isoform abundances; and (D) Correlation between transcript and isoform abundance split by whether there is a unique or ambiguous mapping of peptides onto transcripts/isoforms.

PAQu also provides estimates of the links between transcripts and protein isoforms. Using the same data as described above, we analyze the posterior distributions of the conversion coefficients W (Fig. 1). Interestingly, the distribution of the coefficients related to transcripts and protein isoforms, which are detected as significant in their respective domains, is shifted rightward (Fig. 4A,B). This suggests that in the presence of biological variation in both the transcriptome and the proteome, statistical relationships arise naturally. We decided to investigate this further because the relationship between gene expression measured in tissue and the amount of corresponding protein found in the same tissue has been an intriguing and important scientific puzzle (26, 27). In some experimental settings, gene expression and protein abundance are highly correlated (28–30); in survey studies, the correlation is often low, including most studies evaluating brain tissue (31, 32). While our analyses bring the scale down to RNA transcripts and their protein isoforms, the parallels to previous work are obvious.

Of the 7271 isoforms, 7107 show limited variability between MCMC chains (Methods). For each of these reliably estimated isoforms, we computed the correlation of isoform and transcript abundances over individuals. We obtain a roughly symmetric distribution centered at 0.067 but showing right skew (Fig. 4C). If the true transcript/isoform correlation were zero, then the observed distribution would be normally distributed about zero and only 0.2% would exceed $|\hat{r}|>0.30$, about 7 observations exceeding each bound. Instead, we found 417 transcripts/isoforms with correlations > 0.3, 5.9% of the pairs. We also observed 19 transcripts/isoforms with unusually low correlations, below −0.3 (0.3%). Note that isoforms can be split into two distinct categories: isoforms with a unique mapping of peptides onto isoform (unique) and those for which the measured abundance of a peptide could trace to two or more isoforms (ambiguous).

For the 4056 unique transcripts /isoforms (Fig. 4D), their distribution is centered at 0.087, with 316 (7.8%) in the positive tail and only 6 (0.1%) strongly negative, as previously defined. For the 3051 in the ambiguous set (Fig. 4D), the distribution is centered at 0.041 and it has 101 (3.3%) in the positive tail and 13 (0.4%) in the negative tail. Thus, when there is greater uncertainty in how peptide abundances inform isoform abundances, PAQu is somewhat less accurate, leading to fewer strongly positive correlations.

Next, we focus on extreme positive correlations. As we develop in SI Appendix, Table 1, these correlations must arise from substantial biological variation among samples for both transcripts and isoforms. For example, if cases and controls differed in their means by one half of a standard deviation for both transcript and complementary isoform – an unusually large difference – the expected correlation induced would only be r≈0.053. To determine what variation predicts the observed correlation for each (isoform, transcript) pair, we build a model using the following five predictors: mean case-control difference for isoform/transcript abundances; coefficient for the effect of age on isoform/transcript abundances; and impact of genetic variation on the gene. For this last predictor, we use results from GTEx (33) analysis of gene expression. Specifically, we use the variation of gene expression explained by the eGENE genetic variant (Methods). Using these predictors, we fit the model for two classes of outcomes: the 412 (transcript,isoform) pairs with strong positive correlation values, r>0.3 (5 others did not map uniquely to a single gene); and 412 randomly-chosen (transcript,isoform) pairs, conditional on their correlation being in the range of |r|<0.05. Using all 824 outcomes, the model is significant, with $R^{2}=0.061$ (SI Appendix, Table 1). The impact of age on transcript abundance, diagnosis-based mean isoform differences, and eGENE variation explain a significant portion of the variation. If the low correlation set is analyzed, no predictor explains a significant amount of the variation; when the strong positive correlation set is analyzed, age and diagnosis for both transcripts and isoforms are important predictors, whereas genetic variation is not. The model accounts for a slightly larger portion of the variability of these higher correlation outcomes ($R^{2}=0.092$); SI Appendix, Table 1).

Earlier research (31) found that gene expression within certain gene sets shows higher correlation to resulting protein abundance. To determine if our results show a similar pattern, we next performed a gene ontology overrepresentation analysis (34–36), with the universe set to the genes observed in our data. We queried cellular components (CC) and biological processes (BP). For the genes mapping onto the high correlation set, 40 gene sets show significant enrichment (FDR < 0.05) (SI Appendix, Table 2). Comparing our enriched gene sets with the thirty significantly enriched gene sets from (31), two gene sets overlapped, namely cell surface and extracellular matrix. Given the large set of possible terms for CC and BP, two overlapping gene sets is larger than that expected by chance, and it is perhaps more remarkable because (31) took a very different analytical approach to assess a somewhat different data type.

**Figure 5:**
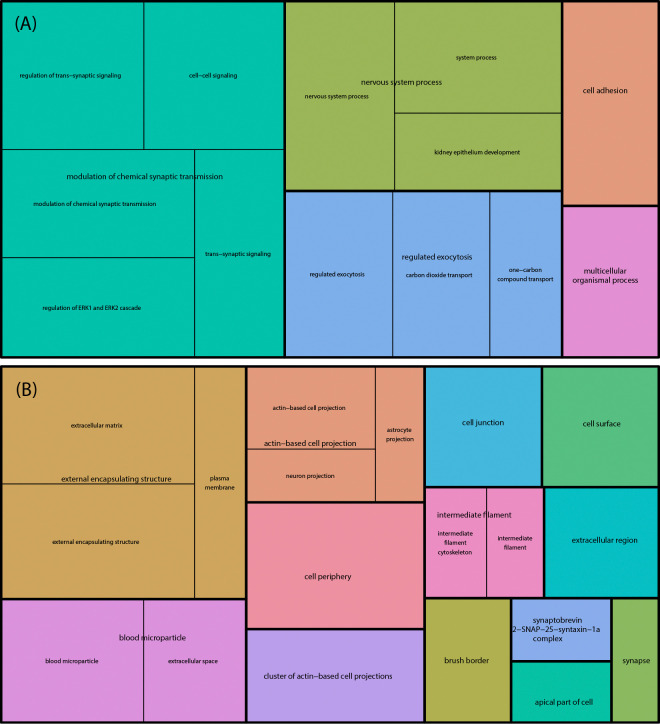
Revigo tree plot summary of enriched Gene Ontology terms for transcripts and isoforms showing large correlaton of abundances. (A) Term enrichment for Biological Processes; (B) Term enrichment for Cellular Component.

We noted a complementary feature of the genes encoding transcripts/isoforms in the high correlation set, namely they were highly enriched for terms related to neuronal and synaptic biology. We used ReviGO (37) to summarize these terms into coherent themes (Fig. 5). This result is consistent with prior studies that show tight scaling of transcription and translation based on synaptic activity (38, 39), although this tight coupling potentially diminishes with increasing age (40, 41).

PAQu also returns estimates of the detectability matrix Z, which projects peptide abundances onto isoform abundances. Peptides vary in their detectability by the mass spectrometer and spectral matching algorithms for a host of reasons, such as their amino acid composition and how those amino acids are chemically altered in vivo, i.e., post-translational modifications (PTMs). For the latter, while the peptide itself has the same amino acid sequence, PTMs change its mass/charge signature and thus the detectability as the same peptide (42). In turn, PTMs can induce variability in measured abundances of peptides from the same isoform and their relationship to the isoform.

## Discussion

Advanced transcriptomics approaches facilitate quantification of transcript alterations in disorders like schizophrenia that could contribute to disease pathology. However, confirming these observations at the isoform level has proven challenging due to bottle necks in proteomic data acquisition and analysis. PAQu stands as an effective tool for addressing several of these challenges: it provides uncertainty quantification for all estimated parameters, integrates external covariate information, elucidates the relationship between the transcriptome and the proteome, highlights the potential presence of post-translational modifications, and allows users to perform differential abundance analysis.

Our analyses of transcriptome and proteome from postmortem dorsal anterior cingulate cortex (dACC) gray matter reveal close integration of certain neuronal and synaptic transcripts and isoforms (Fig. 4, Fig. 5). Meaningful biological variation, including changes in abundance with aging, based on case-status, and genetic variation determine some of this integration (Table 1). For instance, among the 412 highly correlated transcripts and isoforms (r>0.3), 47 are classified under the term regulation of trans-synaptic signaling (GO:0099177), and these show the greatest enrichment for Gene Ontology’s Biological Processes.

Our results on case-control differences for transcripts and isoforms confirm a critical observation made in schizophrenia, first published by Sekar et al. (43) 10 years ago. Elevated levels of the Complement Component 4 transcript C4A, but not C4B, are a genetically based risk factor for schizophrenia, directly correlated to structurally diverse risk alleles. This increase in C4A is widely believed to contribute to impaired cognitive and sensory processing in schizophrenia via its promotion of dendritic spine pruning (44). As these isoforms are almost identical, antibodies cannot distinguish between the two, and thus the findings could not be confirmed at the protein level. Our data captures 38 peptides of C4, 36 mapping to both isoforms and 2 mapping exclusively to C4B. Our results show, for the first time, that both C4A transcript and protein isoform levels are significantly increased in schizophrenia. By contrast, and as predicted by transcript levels, the abundance of C4B did not change with case status. We find that several other transcript alterations with relevance for glial and synaptic processes, implicated together in schizophrenia, are realized at the isoform level. For example, CD44 isoform P16070 is also significantly elevated in cases, which is consistent with recent results showing upregulation of reactive astrocyte marker CD44 in the presence of elevated levels of C4A (45). However, not all synaptic transcript changes result in concurrent isoform alterations, as is the case in CASKIN2, a key molecule for excitatory synaptic function (46). Levels of the CASKIN2 transcript ENST00000321617 are decreased whereas its isoform, Q8WXE0, is significantly elevated, possibly due to a compensatory mechanism to maintain synaptic transmission. Collectively, our findings in schizophrenia demonstrate the power of PAQu to provide confirmation that transcript level alterations manifest at the protein level, providing a direct link from genetic risk to protein function while also showing that not all such changes are translated.

PAQu has limitations; for instance, it can only work in scenarios when q≤r. We envision several possible extensions for our method. First, in situations where the number of samples and the number of isoforms with shared peptides are large, the computational load could be significantly reduced by replacing sampling with a variational inference approach. Second, we would like to investigate the effect of post-translational modifications in greater detail, perhaps by extending PAQu to incorporate information regarding them. Third, we would like to extend PAQu as to integrate data from additional domains other than the transcriptome and peptidome.

In conclusion, in this paper we propose PAQu, a powerful new framework for the analysis of mass spectrometry data that takes advantage of additional information about the transcriptome to quantify the abundance of protein isoforms. By implementing a version of Bayesian supervised factor analysis, we believe that PAQu has the potential to help researchers make full use of mass spectrometry data.

## Materials and Methods

### Notation and PAQu model

We define the n×q matrix of transcript expression data as T, with $T_{ij}$ representing its (i,j)-th element. Similarly, the n×r matrix of peptide abundance data is denoted by P, where $P_{ik}$ is the (i,k)-th element. The vector A is an n×1 binary indicator describing the condition of sample i, with $A_{i}\in \{0,1\}$. The indices i=1,…,n, j=1,…,q, and k=1,…,r refer to the samples, isoforms, and peptides, respectively. Our objective is to infer the parameters that link these quantities, as described by the following model:

$$\begin{array}{l} I=I^{0}+TW+AD^{T}+E^{\text{I}},\\ \;P=IZ+E^{\text{P}}. \end{array}$$

Here, I is an n×q matrix where $I_{ij}$ represents the unobserved abundance of isoform j in sample i, and $I^{0}$ is an n×q matrix of intercepts, with $I_{ij}^{0}$ indicating the baseline abundance of isoform j across samples ($I_{ij}^{0}$ is constant for every i). The diagonal matrix W has elements $W_{jj}$, representing the conversion weight between transcript j and its corresponding protein isoform. The vector D is q×1, where each $D_{j}$ describes the effect of the binary condition A on the abundance of protein isoform j. The matrix Z is a q×r matrix of detectability coefficients, with $Z_{jk}=0$ if peptide k is not associated with isoform j, and otherwise unknown. The terms $E^{\text{I}}$ and $E^{\text{P}}$ are random heteroskedastic Gaussian noise terms affecting isoform and peptide abundances, respectively. Note that knowledge of T and A is not strictly necessary: PAQu can operate as long as P and a detectability mask are provided. We include here these additional components in the model for the sake of completeness. In SI Appendix, Section A, we provide details and in Section B, we describe how to use PAQu in cases where information about T and A is limited or absent, or in the presence of external covariates.

### Estimation and inference with PAQu

#### Prior distributions

The parameters in the model described in Eq. 1 are not identifiable. Thus, standard optimization procedures can lead only to locally optimal solutions. A viable alternative is provided by the Bayesian framework, which requires specification of prior distributions on parameters. We therefore assume that any conversion weight between a transcript j and its protein isoform follows a Gaussian distribution:

(2) $$W_{jj}∼N\left(1,\tau_{j}^{\text{w}}\right),\;j\in \{1,\ldots ,q\},$$

where $\tau_{j}^{\text{w}}$ denotes the prior variance. The prior mean is set to 1, which follows the theoretical assumption of perfect proportionality between transcript expression levels and corresponding protein isoform abundances.

We impose a similar Gaussian prior on condition effects D:

(3) $$D_{j}∼N\left(0,\tau_{j}^{\text{D}}\right),\;j\in \{1,\ldots ,q\},$$

where $\tau_{j}^{\text{D}}$ denotes the prior variance, and the prior mean is set to 0 to reflect conservative prior knowledge about potential differential isoform abundances.

The prior distribution for detectability scores depends on the compatibility between protein isoforms and peptides. We write k≺j if a peptide k is compatible with an isoform j. Therefore, we can write the prior distribution as a mixture between a truncated Gaussian and a Dirac mass at 0:

(4) $$\begin{array}{l} Z_{jk}∼𝟙\{k≺j\}{\bar{N}}_{\bar{l}}^{\bar{u}}\left(1,\tau_{j}^{Z}\right)+(1-𝟙\{k≺j\})\delta_{0},\\ \;j\in \{1,\ldots ,q\},k\in \{1,\ldots ,r\}, \end{array}$$

where $\bar{N}$ denotes a Truncated Normal distribution with prior mean 1, prior variance $\tau_{j}^{\text{Z}}$, and lower and upper truncation parameters $\bar{l}={\bar{P}}_{j}^{min}/{\bar{P}}_{j}^{max}$ and $\bar{u}={\bar{P}}_{j}^{max}/{\bar{P}}_{j}^{min}$ m with ${\bar{P}}_{j}^{min}=\min_{k≺j}n^{-1}\sum_{i=1}^{n}P_{ik}$ and ${\bar{P}}_{j}^{max}=\min_{k≺j}n^{-1}\sum_{i=1}^{n}P_{ik}$. We employ this truncated prior specification for Z to guarantee that estimates in I are consistent to observed values at the peptide level.

We also specify a Gaussian prior for the intercept $I^{0}$:

(5) $$I_{j}^{0}∼N\left(0,\tau_{j}^{{\text{I}}^{0}}\right),\;j\in \{1,\ldots ,q\},$$

where $\tau_{j}^{{\text{I}}^{0}}$ denotes the prior variance. Notice that in an experiment where transcript expression levels have not been measured, but the scientist has access to average expression levels from another study, these may be used as prior means for the intercept (see SI Appendix, Section B for details).

In alternative to the Gaussian prior, PAQu offers the possibility of implementing a “spike-and-slab” prior. For example, the “spike-and-slab” prior for D reads as:

(6) $$D_{j}∼\theta_{j}N\left(0,\tau_{j}^{D}\right)+\left(1-\theta_{j}\right)\delta_{0},\;j\in \{1,\ldots ,q\},$$

where $\theta_{j}$ is the prior probability that a given isoform j is differentially abundant across conditions. As supported by our simulation study, we prefer to use Gaussian priors as they lead to more sensible results.

The last fundamental prior distribution, which links the first and second layers (Fig. 1), is the one for I. Again, we assume a Gaussian prior, this time informing it with knowledge about the other covariates:

(7) $$I_{ij}∼N\left(T_{ij}W_{jj}+A_{i}D_{j},1\right),\;i\in \{1,\ldots ,n\},j\in \{1,\ldots ,q\}.$$

Intuitively, the prior distribution in the previous Equation guarantees that the protein isoforms approximately lie in the space spanned by transcripts. This allows PAQu to supervise the factor analysis of peptides in the second layer in a smooth and unified manner. The prior distributions of the other parameters in the model are specified in SI Appendix, Section A.

#### Posterior sampling

We approximate the posterior distribution of the model parameters using Gibbs sampling, which is a natural choice since we can derive closed-form expressions for the *full conditionals*. Intuitively, given the values of (W, D), I, or Z, the remaining unknown parameters can be easily determined by linear regression. This approach simplifies the complexity of estimating a multivariate posterior distribution by breaking it down into several manageable univariate problems. The analytical posterior distributions of all parameters in the model – using both Gaussian and “spike-and-slab” priors – are specified in the SI Appendix, Section A.

Our choice of default parameters for the Gibbs sampling procedure includes 3000 MCMC iterations and a burn-in period of 2000 iterations. As a result, each MCMC chain approximates its corresponding conditional posterior distribution using 1000 samples.

#### Measuring significance

Although posterior distributions contain all the information about the parameters in the model, we use the Local False Sign Rate (LFSR) proposed by (24) to summarize them in a compact and interpretable value to perform significance hypothesis testing. Intuitively, the LFSR measures confidence in the sign of the effect. We are particularly interested in evaluating when the effect $D_{j}$ of a condition A on an isoform j is significantly different from 0. We thus compute the LFSR as:

(8) $$\text{LFSR}\left(D_{j}\right)=\min\left\{\mathbb{P}\left[D_{j}\geq 0|\cdot \right],\mathbb{P}\left[D_{j}\leq 0|\cdot \right]\right\},$$

where $\mathbb{P}\left[D_{j}\geq 0|\cdot \right]$ and $\mathbb{P}\left[D_{j}\leq 0|\cdot \right]$ denote the posterior probabilities that $D_{j}$ is greater or smaller than 0, respectively. We similarly define the LFSR for the other parameters.

### Simulation study

We simulate transcript, protein isoform, and peptide data under the two-layer linear model in Eq. 1. We simulate data in two different settings. In the first “easy” setting, we generate data without shared peptides. Specifically, we sample one transcript (q=1) from a Normal distribution (i.e. $T_{ij}∼N(3,1)$), map it into a protein isoform $I_{ij}$ with a random conversion weight $\left(W_{jj}∼N(1,0.5)\right)$, add an effect due to a binary condition $\left(D_{j}\in \{0.33,0.66,1\}\right)$, a random intercept term ($I_{ij}^{0}\sim N(10,0.5)$, constant for all i) and random noise $\left(E_{ij}^{I}\sim N(0,1)\right)$, and then split it into two peptides (r=2) using randomly assigned detectability scores $\left(Z_{jk}\sim {\bar{N}}_{-0.5}^{0.5}(1,1)\right)$, again adding random noise $\left(E_{ik}^{\text{P}}\sim N(0,1)\right)$. We define $A_{i}=1$ for the first half of the sample, i.e. for i=1,…,n/2, and $A_{i}=0$ otherwise. In symbols:

(9) $$\begin{array}{l} I_{ij}=I_{ij}^{0}+T_{ij}W_{jj}+A_{i}D_{j}+E_{ij}^{\text{I}},\;i\in \{1,\ldots ,n\},j=q=1,\\ \;P_{ik}=I_{ij}Z_{jk}+{\text{E}}_{ik}^{\text{P}},\;i\in \{1,\ldots ,n\},k\in \{1,r=2\}. \end{array}$$

In the second “difficult” setting, we generate data that include shared peptides. Here, we sample five transcripts (q=5) from a multivariate Normal distribution with identity covariance matrix (i.e. $T_{ij}\sim N(3,1)$), map them to their corresponding protein isoforms $I_{ij}$ with random conversion weights $\left(W_{jj}\sim N(1,0.5)\right)$, add an intercept term $\left(I_{ij}^{0}\sim N(10,0.5)\right)$, and introduce a binary condition effect $\left(D_{j}\in \{0.33,0.66,1\}\right)$ to the first $\left|D^{act}\right|\in \{1,2,3\}$ isoforms, i.e. $D_{j}=D_{j}𝟙\left\{j\leq \left.\left|D^{act}\right|\right\}\right.$. The isoforms are then split into 10 peptides (r=10) using random detectability scores, with s=30% of the detectability matrix entries being nonzero, i.e. $Z_{jk}\sim s{\bar{N}}_{-0.5}^{0.5}(1,1)+(1-s)\delta_{0}$. As before, we define $A_{i}=1$ for the first half of the sample, i.e. for i=1,…,n/2, and $A_{i}=0$ otherwise. In symbols:

(10) $$\begin{array}{l} I_{ij}=I_{ij}^{0}+T_{ij}W_{jj}+A_{i}D_{j}+E_{ij}^{I},\\ \;i\in \{1,\ldots ,n\},j\in \{1,\ldots ,q=5\}, \end{array}$$

(11) $$\begin{array}{l} P_{ik}=\sum_{j=1}^{q}I_{ij}Z_{jk}+E_{ik}^{\text{P}},\\ \;i\in \{1,\ldots ,n\},k\in \{1,\ldots ,r=10\}. \end{array}$$

We run each simulation experiment with three different sample sizes (n=100,200,500), and with 25 different seeds. We run PAQu for 3000 Gibbs sampling iterations, burning in the first 2000 iterations.

### Relationship to other methodology

We want to point out that, from a technical standpoint, our approach shares some similarities with (47, 48), as both employ a variant of Bayesian factor analysis. However, our method differs significantly in several key aspects. Specifically, we adopt different prior distributions, implement a distinct sampling algorithm, and target a different application focus. These differences result in a more tailored and efficient solution to the problem of protein isoform quantification, setting PAQu apart from previous approaches.

Moreover, by developing a tool for supervised factor analysis, our work has some connections to (49–51). However, these works only provide inference for latent factors (in our case, protein isoforms). Instead, PAQu provides estimates for a full class of parameters, shedding light on the biological processes behind protein formation.

#### Sample preparation, LC-MS/MS measurement, and database search for proteins

See In SI Appendix, Section C, for full details. In brief, brain samples were obtained from the University of Pittsburgh brain bank. A tissue sample of similar size was collected from each brain. Each sample was homogenized, its proteins were then solubilized, digested into peptides, TMT labeled, fractionated, and analyzed with an Orbitrap Tribrid Eclipse mass spectrometer. Samples were processed in batches of 14 samples, which will be described as a plex. RNA transcripts observed in the same brains (25), as measured by RNA-seq, were used to inform which isoforms were likely to be found in these samples (isoform matching).

#### Data preprocessing

##### Peptide abundance data.

We first normalize raw expression values of samples to account for differences in total expression levels across samples (see SI Appendix, Section C, for details). After log_2_-transforming normalized values, effects of batch (aka, plex), diagnosis, age, and postmortem interval are modeled using ordinary least squares (OLS) regression. Effects of plex were subtracted from the data, leaving residuals that capture biological variation.

##### Transcript expression data.

Transcript expression data were produced by the CommonMind Consortium (25) and were downloaded from https://www.synapse.org, specifically syn29442530, which was accessed on February 4, 2025. (These data have been moved to the NIMH Data Archive.) We first normalize transcript expression counts, transform them to counts per million, set them on a log_2_ scale, and remove the effect of technical covariates (see SI Appendix, Section C, for details).

### PAQu analysis of schizophrenia dataset

We exploit the block-diagonal structure in Z to divide the original problem into 5241 independent blocks. We then combined isoforms represented by the same set of peptides into isoform groups. This reduces the number of isoforms from 9169 to 7271. Of these, 5831 are singleton isoforms while 3338 isoforms were grouped into 1440 isoform groups with 2 or more isoforms. Out of the 1440 isoform groups, 1396 represented isoforms from a single gene, the remaining 44 represent multiple genes. From here on, we will refer to the combined set of singleton isoforms and isoform groups as isoforms.

### GO enrichment analysis

We perform GO enrichment analysis using the g:GOSt tool for functional profiling contained in the g:profiler toolkit (52), version *e*111_*eg*58_*p*18_*f* 463989*d*, with Benjamini-Hochberg significance threshold set at 0.05. All ENSG isoforms employed in the analysis are treated as background; isoforms with LFSR ≤ 0.05 are treated as foreground.

### Analysis of transcript and complementary isoform correlations

Of the 7271 isoforms, we selected for analysis those isoforms with low variability of estimated abundance across MCMC chains. Specifically, for each isoform and MCMC chain, we computed the mean abundance over 103 samples. Next, we computed the variance of the mean over the 10 MCMC chains. If the variance fell below one, the isoform was retained (N=7107); otherwise, it was removed (N=164). We then calculated the Pearson correlation, over samples, for each of the 7107 transcript/isoform pairs. To predict the observed correlation of transcript/isoform pairs, we estimated the effects for diagnosis and age from the estimated isoform abundances and adjusted transcript expression. It is well known that gene expression and protein abundance can vary by genotype. To quantify the impact of genetic variation, we gathered information on 26,134 eGenes for the cortex from the GTEx website (53). (By definition, an eGene is the genetic variant that explains the greatest variance in gene expression.) From this set, we selected 16,491 protein-coding genes and recorded the estimate of the slope (b) and allele frequency (f). The estimated variance explained by each eGene was then determined as 2f $(1-f)b^{2}$, and this value was used to predict the observed correlation of transcript/isoform pairs. Transcript/isoform groups mapping to multiple genes were excluded from this analysis.

## Supplementary Material

Supplement 1
