# Supplementary material for: Estimating protein isoform abundances with PAQu
Source: bioRxiv. 2026 Apr 22:2026.04.20.719668. Preprint. [Version 1] doi: 10.64898/2026.04.20.719668 (PMC13131628; doi:10.64898/2026.04.20.719668)
Supplement: Supplement 1 [file NIHPP2026.04.20.719668v1-supplement-1.pdf]

Supplementary Information

A Main PAQu model specification

Priors

Here, we provide the complete list of prior distributions employed in our main model specification:

$$\mathbf{I}^{\mathbf{0}}_j \sim \mathcal{N}\left(0, \tau^{\mathbf{I}^0}_j\right), \quad j \in \{1, \ldots, q\}, \tag{12}$$

$$\mathbf{W}_{jj} \sim \mathcal{N}\left(1, \tau^{\mathbf{W}}_j\right), \quad j \in \{1, \ldots, q\}, \tag{13}$$

$$\mathbf{D}_j \sim \mathcal{N}\left(0, \tau^{\mathbf{D}}_j\right), \quad j \in \{1, \ldots, q\}, \tag{14}$$

$$\mathbf{I}_{ij} \sim \mathcal{N}\left(\mathbf{I}^{\mathbf{0}}_j + \mathbf{T}_{ij}\mathbf{W}_{jj} + \mathbf{A}_i\mathbf{D}_j, 1\right), \quad i \in \{1, \ldots, n\}, j \in \{1, \ldots, q\}, \tag{15}$$

$$\tau^{\mathbf{I}^0}_j \sim \text{InvGamma}(1, 1), \quad j \in \{1, \ldots, q\}, \tag{16}$$

$$\tau^{\mathbf{W}}_j \sim \text{InvGamma}(1, 1), \quad j \in \{1, \ldots, q\}, \tag{17}$$

$$\tau^{\mathbf{D}}_j \sim \text{InvGamma}(3, 0.5), \quad j \in \{1, \ldots, q\}, \tag{18}$$

$$\sigma^{\mathbf{I}}_j \sim \text{InvGamma}(1, 1), \quad j \in \{1, \ldots, q\}, \tag{19}$$

$$\mathbf{Z}_{jk} \sim \mathbb{1}\{k \prec j\}\mathcal{N}^{\bar{u}}_{\bar{l}}\left(1, \tau^{Z}_j\right) + (1 - \mathbb{1}\{k \prec j\})\delta_0, \quad j \in \{1, \ldots, q\}, k \in \{1, \ldots, r\}, \tag{20}$$

$$\tau^{\mathbf{Z}}_j \sim \text{InvGamma}(1, 1), \quad j \in \{1, \ldots, q\}, \tag{21}$$

$$\sigma^{\mathbf{P}}_k \sim \text{InvGamma}(1, 1), \quad k \in \{1, \ldots, r\}. \tag{22}$$

Posteriors

We exploit conjugacy in the Bayesian framework to obtain the following *full conditional* posteriors:

$$\mathbf{I}^{\mathbf{0}}_j \,|\, \cdot \sim \mathcal{N}\left(\frac{(\sigma^{\mathbf{I}}_j)^{-1}\sum_{i=1}^n(\mathbf{I}_{ij} - \mathbf{T}_{ij}\mathbf{W}_{jj} - \mathbf{A}_i\mathbf{D}_j)}{(\tau^{\mathbf{I}^0}_j)^{-1} + (\sigma^{\mathbf{I}}_j)^{-1}n}, \frac{1}{(\tau^{\mathbf{I}^0}_j)^{-1} + (\sigma^{\mathbf{I}}_j)^{-1}n}\right), \quad j \in \{1, \ldots, q\}, \tag{23}$$

$$\mathbf{W}_{jj} \,|\, \cdot \sim \mathcal{N}\left(\frac{(\tau^{\mathbf{W}}_j)^{-1} + (\sigma^{\mathbf{I}}_j)^{-1}\sum_{i=1}^n(\mathbf{I}_{ij} - \mathbf{I}^{\mathbf{0}}_j - \mathbf{A}_i\mathbf{D}_j)\mathbf{T}_{ij}}{(\tau^{\mathbf{W}}_j)^{-1} + (\sigma^{\mathbf{I}}_j)^{-1}\sum_{i=1}^n\mathbf{T}^2_{ij}}, \frac{1}{(\tau^{\mathbf{W}}_j)^{-1} + (\sigma^{\mathbf{I}}_j)^{-1}\sum_{i=1}^n\mathbf{T}^2_{ij}}\right), \quad j \in \{1, \ldots, q\}, \tag{24}$$

$$\mathbf{D}_j \,|\, \cdot \sim \mathcal{N}\left(\frac{(\sigma^{\mathbf{I}}_j)^{-1}\sum_{i=1}^n(\mathbf{I}_{ij} - \mathbf{I}^{\mathbf{0}}_j - \mathbf{T}_{ij}\mathbf{W}_{jj})\mathbf{A}_i}{(\tau^{\mathbf{D}}_j)^{-1} + (\sigma^{\mathbf{I}}_j)^{-1}\sum_{i=1}^n\mathbf{A}^2_i}, \frac{1}{(\tau^{\mathbf{D}}_j)^{-1} + (\sigma^{\mathbf{I}}_j)^{-1}\sum_{i=1}^n\mathbf{A}^2_i}\right), \quad j \in \{1, \ldots, q\}, \tag{25}$$

$$\mathbf{I}_{ij} \,|\, \cdot \sim \mathcal{N}\left(\frac{\mathbf{I}^{\mathbf{0}}_j + \mathbf{T}_{ij}\mathbf{W}_{jj} + \mathbf{A}_i\mathbf{D}_j + \sum_{k=1}^r(\mathbf{P}_{ik} - \sum_{c\neq j}\mathbf{I}_{ic}\mathbf{Z}_{ck})\mathbf{Z}_{jk}(\sigma^{\mathbf{P}}_k)^{-1}}{(\tau^{\mathbf{I}}_j)^{-1} + \sum_{k=1}^r\mathbf{Z}^2_{jk}(\sigma^{\mathbf{P}}_k)^{-1}}, \frac{1}{(\tau^{\mathbf{I}}_j)^{-1} + \sum_{k=1}^r\mathbf{Z}^2_{jk}(\sigma^{\mathbf{P}}_k)^{-1}}\right),$$
$$i \in \{1, \ldots, n\}, j \in \{1, \ldots, q\}, \tag{26}$$

bioRxiv preprint doi: https://doi.org/10.64898/2026.04.20.719668; this version posted April 22, 2026. The copyright holder for this preprint (which was not certified by peer review) is the author/funder, who has granted bioRxiv a license to display the preprint in perpetuity. It is made available under aCC-BY 4.0 International license.

$$\tau_j^{\mathbf{I^0}} \,|\, \cdot \sim \text{InvGamma}\left(\frac{3}{2}, 1 + \frac{(\mathbf{I}_j^{\mathbf{0}})^2}{2}\right), \quad j \in \{1, \dots, q\}, \tag{27}$$

$$\tau_j^{\mathbf{W}} \,|\, \cdot \sim \text{InvGamma}\left(\frac{3}{2}, 1 + \frac{\mathbf{W}_{jj}^2}{2}\right), \quad j \in \{1, \dots, q\}, \tag{28}$$

$$\tau_j^{\mathbf{D}} \,|\, \cdot \sim \text{InvGamma}\left(\frac{3}{2}, 1 + \frac{\mathbf{D}_j^2}{2}\right), \quad j \in \{1, \dots, q\}, \tag{29}$$

$$\sigma_j^{\mathbf{I}} \,|\, \cdot \sim \text{InvGamma}\left(1 + \frac{n}{2}, 1 + \frac{\sum_{i=1}^n (\mathbf{I}_{ij} - \mathbf{I}_j^{\mathbf{0}} - \mathbf{T}_{ij}\mathbf{W}_{jj} - \mathbf{A}_i \mathbf{D}_j)^2}{2}\right), \quad j \in \{1, \dots, q\}, \tag{30}$$

$$\mathbf{Z}_{jk} \,|\, \cdot \propto \mathbb{1}\{k \prec j\} \mathcal{N}_{\bar{l}}^{\bar{u}}\left(\frac{(\tau_j^{\mathbf{Z}})^{-1} + (\sigma_k^{\mathbf{P}}) \sum_{i=1}^n (\mathbf{P}_{ik} - \sum_{c \neq j} \mathbf{I}_{ic}\mathbf{Z}_{ck}) I_{ij}}{(\tau_j^{\mathbf{Z}})^{-1} + (\sigma_k^{\mathbf{P}})^{-1} \sum_{i=1}^n \mathbf{I}_{ij}^2}, \frac{1}{(\tau_j^{\mathbf{Z}})^{-1} + (\sigma_k^{\mathbf{P}})^{-1} \sum_{i=1}^n \mathbf{I}_{ij}^2}\right) + (1 - \mathbb{1}\{k \prec j\})\delta_0, \quad j \in \{1, \dots, q\}, k \in \{1, \dots, r\}, \tag{31}$$

$$\tau_j^{\mathbf{Z}} \,|\, \cdot \sim \text{InvGamma}\left(1 + \frac{\mathbb{1}\{k \prec j\}}{2}, 1 + \frac{\sum_{k=1}^r \mathbf{Z}_{jk}^2}{2}\right), \quad j \in \{1, \dots, q\}, \tag{32}$$

$$\sigma_k^{\mathbf{P}} \,|\, \cdot \sim \text{InvGamma}\left(1 + \frac{n}{2}, 1 + \frac{\sum_{i=1}^n (\mathbf{P}_{ik} - \sum_{j=1}^q \mathbf{I}_{ij}\mathbf{Z}_{jk})^2}{2}\right), \quad k \in \{1, \dots, r\}. \tag{33}$$

Pseudocode and algorithmic implementation

Parameter initialization. We initialize $\mathbf{I}$ and $\mathbf{Z}$ from a truncated singular value decomposition (SVD) of $\mathbf{P}$. Elements in $\mathbf{Z}$ are then masked according to the mask matrix $\mathbf{M}$. We initialize $\mathbf{I^0}$, $\mathbf{D}$, and $\mathbf{W}$ using ordinary least squares (OLS) estimates. In particular, we jointly regress each isoform on the corresponding transcript, the unit constant vector, and the condition vector $\mathbf{A}$. All the other hyperparameters are originally initialized at 1.

Parallelization. By exploiting the sparsity in $\mathbf{Z}$, we can divide the original problem into smaller, much easier-to-solve subproblems. In particular, $\mathbf{Z}$ presents a block-diagonal structure up to a permutation. Therefore, we only need to focus on one diagonal block at a time. Each diagonal block involves only the isoforms that share at least one peptide. In other words, isoforms with independent peptides can be analyzed separately. As a result, we can parallelize the computation block-by-block.

Pseudocode. Algorithm 1 below contains the pseudocode of `PAQu` running on an individual diagonal block.

Algorithm 1 `PAQu` algorithm pseudocode

```
Input: T, A, P, M, S (number of Gibbs iterations)
Output: approximate posterior distributions, LFSR and means of I^0, W, D, I, Z, and hyperparameters
Initialize unknown quantities I^0(0), W(0), D(0), I(0), Z(0), and hyperparameters
for s ← 1 to S do
    Sample I^0(s), W(s), D(s), I(s), Z(s), and hyperparameters
end for
Estimate posterior means and compute LFSR from approximate posterior distributions
```

bioRxiv preprint doi: https://doi.org/10.64898/2026.04.20.719668; this version posted April 22, 2026. The copyright holder for this preprint (which was not certified by peer review) is the author/funder, who has granted bioRxiv a license to display the preprint in perpetuity. It is made available under aCC-BY 4.0 International license.

Spike-and-slab implementation

The prior structure of the problem changes only slightly. In particular, we need to introduce the following prior distributions:

$$\mathbf{D}_j \sim \theta_j \mathcal{N}\left(0, \tau_j^{\mathbf{D}}\right) + (1-\theta_j)\delta_0\,, \quad j \in \{1,\ldots,q\}\,, \tag{34}$$

$$\theta_j \sim \text{Beta}(1,1)\,, \quad j \in \{1,\ldots,q\}\,, \tag{35}$$

$$\pi_j \sim \text{Bernoulli}(\theta_j)\,, \quad j \in \{1,\ldots,q\}\,. \tag{36}$$

The derivation of the posterior distribution of π is not trivial. We first compute the log odds y (for numerical stability):

$$y_j = \frac{1}{2}\log\left(\frac{1}{\tau_j^{\mathbf{D}}((\tau_j^{\mathbf{D}})^{-1} + \sum_{i=1}^n \mathbf{A}_i^2)}\right) + \frac{\sum_{i=1}^n ((\mathbf{I}_{ij} - \mathbf{I}_j^{\mathbf{0}} - \mathbf{T}_{ij}\mathbf{W}_{jj})\mathbf{A}_i)^2}{2((\tau_j^{\mathbf{D}})^{-1} + \sum_{i=1}^n \mathbf{A}_i^2)} + \log(\theta_j) - \log(1-\theta_j)\,. \tag{37}$$

We then evaluate its logit transformation $\tilde{\pi}_j = 1/(1+\exp(-y_j))$. We thus sample from the posterior of π_j by computing $\pi_j \,|\, \cdot \sim \text{Bernoulli}(\tilde{\pi}_j)$.

The posterior distribution of θ is given by:

$$\theta_j \,|\, \cdot \sim \text{Beta}(1+\pi_j, 1+(1-\pi_j))\,, \quad j \in \{1,\ldots,q\}\,. \tag{38}$$

Finally, the posterior distribution of $\mathbf{D}_j$ is as before if $\pi_j = 1$; a Dirac mass at 0 otherwise.

B Alternative `PAQu` model specifications

The minimal requirement for `PAQu` to run are the peptide abundance matrix and the mask matrix. Here, we describe several extensions of the model described in the main manuscript.

Absent information on transcripts or condition

In some scenarios, one may not have access to information on transcript expression levels or binary conditions. In these cases, `PAQu` operates by setting $\mathbf{W} = 0$ and $\mathbf{D} = 0$, respectively. Notice that, if information on transcripts is absent, then the user has to specify the number of protein isoform factors to employ to perform factor analysis. Therefore, in this case, q must be set in advance.

Limited information on transcripts

In some experiments, transcript expression levels may not have been measured, but the scientist has access to average expression levels from another study. In this case, these may be used as prior means for the intercept $\mathbf{I^0}$. In particular, define the average expression level for transcript j as μ_j. Then the prior distribution for $\mathbf{I^0}$ is:

$$\mathbf{I}_j^{\mathbf{0}} \sim \mathcal{N}\left(\mu_j, \tau_j^{\mathbf{I^0}}\right)\,, \quad j \in \{1,\ldots,q\}\,. \tag{39}$$

External covariates

In other settings, one may have access to additional information about protein isoforms or peptides. `PAQu` can accomodate external covariates in a straightforward manner. First, define $\mathbf{X^I}$ and $\mathbf{X^P}$ as the $n \times p^I$ and $n \times p^P$ matrices of external covariates that provide information on protein isoforms and peptides, respectively. We incorporate them into the model in Eq. 1:

$$\begin{aligned} \mathbf{I} &= \mathbf{I^0} + \mathbf{TW} + \mathbf{AD}^T + \mathbf{X^I B^I} + \mathbf{E^I}\,, \\ \mathbf{P} &= \mathbf{IZ} + \mathbf{X^P B^P} + \mathbf{E^P}\,. \end{aligned} \tag{40}$$

bioRxiv preprint doi: https://doi.org/10.64898/2026.04.20.719668; this version posted April 22, 2026. The copyright holder for this preprint (which was not certified by peer review) is the author/funder, who has granted bioRxiv a license to display the preprint in perpetuity. It is made available under aCC-BY 4.0 International license.

Here, the matrices $\mathbf{B^I}$ and $\mathbf{B^P}$ are $p^I \times q$ and $p^P \times r$, capturing the effects of external covariates $\mathbf{X^I}$ and $\mathbf{X^P}$ on isoforms and peptides. The interpretation of the other variables is the same as before.

In this new setting, we need to define the following prior distributions:

$$\mathbf{I}_{ij} \sim \mathcal{N}\left(\mathbf{T}_{ij}\mathbf{W}_{jj} + \mathbf{A}_i\mathbf{D}_j + \sum_{\ell=1}^{p^I} \mathbf{X}^{\mathbf{I}}_{i\ell}\mathbf{B}^{\mathbf{I}}_{\ell j}, 1\right), \quad i \in \{1,\ldots,n\}, j \in \{1,\ldots,q\}, \tag{41}$$

$$\mathbf{B}^{\mathbf{I}}_{\ell j} \sim \mathcal{N}\left(0, \tau^{\mathbf{B^I}}_{\ell}\right), \quad \ell \in \{1,\ldots,p^I\}, j \in \{1,\ldots,q\}, \tag{42}$$

$$\mathbf{B}^{\mathbf{P}}_{\ell k} \sim \mathcal{N}\left(0, \tau^{\mathbf{B^P}}_{\ell}\right), \quad \ell \in \{1,\ldots,p^P\}, k \in \{1,\ldots,r\}, \tag{43}$$

$$\tau^{\mathbf{B^I}}_{\ell} \,|\cdot \sim \mathrm{InvGamma}(1,1), \quad \ell \in \{1,\ldots,p^I\}, \tag{44}$$

$$\tau^{\mathbf{B^P}}_{\ell} \sim \mathrm{InvGamma}(1,1), \quad \ell \in \{1,\ldots,p^P\}. \tag{45}$$

By similar computations as above, we get the following posteriors:

$$\mathbf{I}^{\mathbf{0}}_j \,|\cdot \sim \mathcal{N}\left(\frac{(\sigma^{\mathbf{I}}_j)^{-1}\sum_{i=1}^n(\mathbf{I}_{ij} - \mathbf{T}_{ij}\mathbf{W}_{jj} - \mathbf{A}_i\mathbf{D}_j - \sum_{\ell=1}^{p^I}\mathbf{X}^{\mathbf{I}}_{i\ell}\mathbf{B}^{\mathbf{I}}_{\ell j})}{(\tau^{\mathbf{I^0}}_j)^{-1} + (\sigma^{\mathbf{I}}_j)^{-1}n}, \frac{1}{(\tau^{\mathbf{I^0}}_j)^{-1} + (\sigma^{\mathbf{I}}_j)^{-1}n}\right), \quad j \in \{1,\ldots,q\}, \tag{46}$$

$$\mathbf{W}_{jj} \,|\cdot \sim \mathcal{N}\left(\frac{(\tau^{\mathbf{W}}_j)^{-1} + (\sigma^{\mathbf{I}}_j)^{-1}\sum_{i=1}^n(\mathbf{I}_{ij} - \mathbf{I}^{\mathbf{0}}_j - \mathbf{A}_i\mathbf{D}_j - \sum_{\ell=1}^{p^I}\mathbf{X}^{\mathbf{I}}_{i\ell}\mathbf{B}^{\mathbf{I}}_{\ell j})\mathbf{T}_{ij}}{(\tau^{\mathbf{W}}_j)^{-1} + (\sigma^{\mathbf{I}}_j)^{-1}\sum_{i=1}^n\mathbf{T}^2_{ij}}, \frac{1}{(\tau^{\mathbf{W}}_j)^{-1} + (\sigma^{\mathbf{I}}_j)^{-1}\sum_{i=1}^n\mathbf{T}^2_{ij}}\right), \quad j \in \{1,\ldots,q\}, \tag{47}$$

$$\mathbf{B}^{\mathbf{I}}_{\ell j} \,|\cdot \sim \mathcal{N}\left(\frac{(\sigma^{\mathbf{I}}_j)^{-1}\sum_{i=1}^n(\mathbf{I}_{ij} - \mathbf{I}^{\mathbf{0}}_j - \mathbf{T}_{ij}\mathbf{W}_{jj} - \mathbf{A}_i\mathbf{D}_j - \sum_{c\neq j}\mathbf{X}^{\mathbf{I}}_{ic}\mathbf{B}^{\mathbf{I}}_{cj})\mathbf{X}^{\mathbf{I}}_{i\ell}}{(\tau^{\mathbf{B^I}}_{\ell})^{-1} + (\sigma^{\mathbf{I}}_j)^{-1}\sum_{i=1}^n(\mathbf{X}^{\mathbf{I}}_{i\ell})^2}, \frac{1}{(\tau^{\mathbf{B^I}}_{\ell})^{-1} + (\sigma^{\mathbf{I}}_j)^{-1}\sum_{i=1}^n(\mathbf{X}^{\mathbf{I}}_{i\ell})^2}\right),$$
$$\ell \in \{1,\ldots,p^I\}, j \in \{1,\ldots,q\}, \tag{48}$$

$$\mathbf{D}_j \,|\cdot \sim \mathcal{N}\left(\frac{(\sigma^{\mathbf{I}}_j)^{-1}\sum_{i=1}^n(\mathbf{I}_{ij} - \mathbf{I}^{\mathbf{0}}_j - \mathbf{T}_{ij}\mathbf{W}_{jj} - \sum_{\ell=1}^{p^I}\mathbf{X}^{\mathbf{I}}_{i\ell}\mathbf{B}^{\mathbf{I}}_{\ell j})\mathbf{A}_i}{(\tau^{\mathbf{D}}_j)^{-1} + (\sigma^{\mathbf{I}}_j)^{-1}\sum_{i=1}^n\mathbf{A}^2_i}, \frac{1}{(\tau^{\mathbf{D}}_j)^{-1} + (\sigma^{\mathbf{I}}_j)^{-1}\sum_{i=1}^n\mathbf{A}^2_i}\right), \quad j \in \{1,\ldots,q\}, \tag{49}$$

$$\mathbf{I}_{ij} \,|\cdot \sim \mathcal{N}\left(\frac{\mathbf{I}^{\mathbf{0}}_j + \mathbf{T}_{ij}\mathbf{W}_{jj} + \mathbf{A}_i\mathbf{D}_j + \sum_{\ell=1}^{p^I}\mathbf{X}^{\mathbf{I}}_{i\ell}\mathbf{B}^{\mathbf{I}}_{\ell j} + \sum_{k=1}^r(\mathbf{P}_{ik} - \sum_{c\neq j}\mathbf{I}_{ic}\mathbf{Z}_{ck} - \sum_{\ell=1}^{p^P}\mathbf{X}^{\mathbf{P}}_{i\ell}\mathbf{B}^{\mathbf{P}}_{\ell k})\mathbf{Z}_{jk}(\sigma^{\mathbf{P}}_k)^{-1}}{(\tau^{\mathbf{I}}_j)^{-1} + \sum_{k=1}^r\mathbf{Z}^2_{jk}(\sigma^{\mathbf{P}}_k)^{-1}}, \frac{1}{(\tau^{\mathbf{I}}_j)^{-1} + \sum_{k=1}^r\mathbf{Z}^2_{jk}(\sigma^{\mathbf{P}}_k)^{-1}}\right),$$
$$i \in \{1,\ldots,n\}, j \in \{1,\ldots,q\}, \tag{50}$$

$$\tau^{\mathbf{B^I}}_{\ell} \,|\cdot \sim \mathrm{InvGamma}\left(1 + \frac{p^I}{2}, 1 + \frac{\sum_{j=1}^q(\mathbf{B}^{\mathbf{I}}_{\ell j})^2}{2}\right), \quad \ell \in \{1,\ldots,p^I\}, \tag{51}$$

$$\sigma^{\mathbf{I}}_j \,|\cdot \sim \mathrm{InvGamma}\left(1 + \frac{n}{2}, 1 + \frac{\sum_{i=1}^n(\mathbf{I}_{ij} - \mathbf{I}^{\mathbf{0}}_j - \mathbf{T}_{ij}\mathbf{W}_{jj} - \mathbf{A}_i\mathbf{D}_j - \sum_{\ell=1}^{p^I}\mathbf{X}^{\mathbf{I}}_{i\ell}\mathbf{B}^{\mathbf{I}}_{\ell j})^2}{2}\right), \quad j \in \{1,\ldots,q\}, \tag{52}$$

bioRxiv preprint doi: https://doi.org/10.64898/2026.04.20.719668; this version posted April 22, 2026. The copyright holder for this preprint (which was not certified by peer review) is the author/funder, who has granted bioRxiv a license to display the preprint in perpetuity. It is made available under aCC-BY 4.0 International license.

$$\mathbf{Z}_{jk} \mid \cdot \propto \mathbb{1}\{k \prec j\} \mathcal{N}_{\bar{l}}^{\bar{u}} \left(\frac{(\tau_j^{\mathbf{Z}})^{-1} + (\sigma_k^{\mathbf{P}}) \sum_{i=1}^{n} (\mathbf{P}_{ik} - \sum_{c \neq j} \mathbf{I}_{ic} \mathbf{Z}_{ck} - \sum_{\ell=1}^{p^P} \mathbf{X}_{i\ell}^{\mathbf{P}} \mathbf{B}_{\ell k}^{\mathbf{P}}) I_{ij}}{(\tau_j^{\mathbf{Z}})^{-1} + (\sigma_k^{\mathbf{P}})^{-1} \sum_{i=1}^{n} \mathbf{I}_{ij}^2}, \frac{1}{(\tau_j^{\mathbf{Z}})^{-1} + (\sigma_k^{\mathbf{P}})^{-1} \sum_{i=1}^{n} \mathbf{I}_{ij}^2} \right) + (1 - \mathbb{1}\{k \prec j\}) \delta_0 , \quad j \in \{1, \dots, q\}, \, k \in \{1, \dots, r\}, \tag{53}$$

$$\mathbf{B}_{\ell k}^{\mathbf{P}} \mid \cdot \sim \mathcal{N} \left(\frac{(\sigma_k^{\mathbf{P}})^{-1} \sum_{i=1}^{n} (\mathbf{P}_{ik} - \sum_{j=1}^{q} \mathbf{I}_{ij} \mathbf{Z}_{jk} - \sum_{c \neq \ell} \mathbf{X}_{ic}^{\mathbf{P}} \mathbf{B}_{ck}^{\mathbf{P}}) \mathbf{X}_{i\ell}^{\mathbf{P}}}{(\tau_\ell^{\mathbf{B}^{\mathbf{P}}})^{-1} + (\sigma_k^{\mathbf{P}})^{-1} \sum_{i=1}^{n} (\mathbf{X}_{i\ell}^{\mathbf{P}})^2}, \frac{1}{(\tau_\ell^{\mathbf{B}^{\mathbf{P}}})^{-1} + (\sigma_k^{\mathbf{P}})^{-1} \sum_{i=1}^{n} (\mathbf{X}_{i\ell}^{\mathbf{P}})^2} \right) \quad \ell \in \{1, \dots, p^P\}, \, k \in \{1, \dots, r\}, \tag{54}$$

$$\tau_\ell^{\mathbf{B}^{\mathbf{P}}} \mid \cdot \sim \text{InvGamma} \left(1 + \frac{p^P}{2}, 1 + \frac{\sum_{k=1}^{r} (\mathbf{B}_{\ell k}^{\mathbf{P}})^2}{2} \right), \quad \ell \in \{1, \dots, p^P\}, \tag{55}$$

$$\sigma_k^{\mathbf{P}} \mid \cdot \sim \text{InvGamma} \left(1 + \frac{n}{2}, 1 + \frac{\sum_{i=1}^{n} (\mathbf{P}_{ik} - \sum_{j=1}^{q} \mathbf{I}_{ij} \mathbf{Z}_{jk} - \sum_{\ell=1}^{p^P} \mathbf{X}_{i\ell}^{\mathbf{P}} \mathbf{B}_{\ell k}^{\mathbf{P}})^2}{2} \right), \quad k \in \{1, \dots, r\}. \tag{56}$$

Collapsed isoforms

In some instances where two or more isoforms share exactly the same peptides and transcript expression levels do not bring any relevant prior information, PAQu may provide unstable estimates across replicate chains. Therefore, we also implement a *collapsed isoforms* strategy where we estimate the abundance of identical isoforms as they were a single meta-isoform. We take the average of their transcript expression levels as prior, if available. This allows PAQu to provide stable estimates of meta-isoforms abundance levels, which can still carry valuable biological insights.

C Isoform and Transcript Characterization in Brain

Processing of Protein

Human tissue sample collection and preparation

Human brain specimens were obtained during autopsies conducted at the Allegheny County Office of the Medical Examiner after receiving consent from the next-of-kin. All procedures were approved by the Committee for the Oversight of Research and Clinical Training Involving Decedents and the Institutional Review Board for Biomedical Research, University of Pittsburgh, Pittsburgh, Pennsylvania. A subset of previously characterized Sz (n = 56) and control subjects (n = 56) were selected (*25*). When possible, subjects were organized into pairs matched by age, sex, and PMI. An independent panel of experienced clinicians made consensus diagnoses foloowing Diagnostic and Statistical Manual of Mental Disorders Fourth Edition (DSM-IV) or their absence using a previously described method. For area identification and blocking, grey matter was collected from the dACC by taking 40μm sections and stored frozen at -80°C. Experimenters were blinded to case-condition during sample preparation. SZ and CT pairs were kept together during all sample preparation steps. Samples were randomized and distributed in a block design for preparation and mass spectrometry analysis.

20mg of dACC grey matter was added to 10% SDS with 1x Protease (0.2%) and Phosphatase (0.5%) Inhibitors in a 1.5mL tube with 20mg of 0.2mm stainless steel beads. Samples were homogenized in a Bullet Blender Homogenizer (Next Advance) at speed 8 for 3 minutes, then supernatant diluted to 5x with (10%) SDS with 1x Protease (0.2%) and Phosphatase (0.5%)

bioRxiv preprint doi: https://doi.org/10.64898/2026.04.20.719668; this version posted April 22, 2026. The copyright holder for this preprint (which was not certified by peer review) is the author/funder, who has granted bioRxiv a license to display the preprint in perpetuity. It is made available under aCC-BY 4.0 International license.

Inhibitors (Sigma, Cat. No., I3786, P5726, P0044) and frozen. Total protein concentration was assessed by micro-BCA (Thermo Fisher, Cat. No., 23235).

S-trap micro (Protifi, Cat. No. C02-micro-80) was used to digest proteins into tryptic peptides. For each sample, 100 µg of total protein was diluted to 50 µl in 5% SDS, 50 mM TEAB buffer, reduced with 5.6 µl 200 mM dithiothreitol (DTT) at 95°C for 10 min, cooled down for 10 min at room temperature (RT), and alkylated with 5.6 µl 400 mM iodoacetamide (IAA) at RT for 30 min. Samples were then acidified with 8.6 µl 12% phosphoric acid, mixed with 569 µl loading/wash buffer (90% methanol, 100 mM TEAB pH 7.1), and loaded into S-trap 96-well plate columns by centrifugation at 1,500×g. Trapped proteins were washed with 200 µl wash buffer and centrifuged at 1,500×g for 1 min at 20°C a total of three times. Proteins were then digested on-column with 10 µg trypsin in 125 µl 50 mM TEAB for 60 min at 47°C. Peptides were collected by sequential centrifugation at 1,500×g for 2 min at 20°C in 125 µl 50 mM TEAB, 125 µl 0.2% formic acid (FA), and 125 µl 50% acetonitrile (ACN). Eluted peptides were combined and dried in a vacuum concentrator.

A pooled control was made from 15 µg aliquots from each sample, which was then split into four aliquots for digestion on S-trap midi columns (Protifi). Samples were reduced with 27.7 µl 200 mM DTT at 95°C for 10 min, cooled down for 10 min at RT, and alkylated with 27.7 µl 400 mM IAA at RT for 30 min. They were then acidified with 30.5 µl 12% phosphoric acid, mixed with 2016 µl loading/wash buffer, and loaded into S-trap columns by centrifugation at 4,000×g. Trapped proteins were washed with 600 µl wash buffer, centrifuged at 4,000×g for 1 min at 20°C three times, and digested on-column with 25 µg trypsin in 125 µl 50 mM TEAB for 60 min at 47°C. Peptides were collected by sequential centrifugation at 4,000×g for 1 min at 20°C in 500 µl 50 mM TEAB, 500 µl 0.2% formic acid (FA), and 500 µl 50% acetonitrile (ACN). Eluted peptides were combined into one tube and dried in a vacuum concentrator.

TMT labeling and High pH reverse phase fractionation

Peptide digests were resuspended in 100 µl 100 mM TEAB and labeled with 40 µl of 10 µg/µl TMTpro reagent in ACN at room temperature (RT) for 1 hr, then quenched with 2.3 µl 5% hydroxylamine for 15 min. Pooled control peptides were split into two aliquots, each resuspended in 1300 µl 100 mM TEAB and labeled with 400 µl of 10 µg/µl TMTpro reagent in ACN at RT for 1 hr. The reaction was quenched with 92.79 µl 5% hydroxylamine for 15 min. A total of 100 µg from each sample was labeled with channels 1–16 in each plex (TMT channels 126–133N), and combined along with 100 µg pooled control peptides labeled with channels 17 and 18 (TMT 134C–135N), resulting in 16 samples and 2 pooled controls per plex. TMT-labeled peptide plexes were dried in a vacuum concentrator and fractionated by offline high-performance liquid chromatography (HPLC).

800 µg from each plex was resuspended in reagent A (4.5 mM ammonium formate, 2% ACN) and loaded onto a Zorbax Extend-C18 Rapid Resolution column (4.6 × 250 mm, 3.5 µm) on a Vanquish Flex system (Thermo Fisher). Samples were eluted with a 109 min gradient of mobile phase reagent B (4.5 mM ammonium formate, 90% ACN) at a flow rate of 0.800 mL/min. The gradient program was as follows: 0% B for 13 min, increasing to 16% B over 60 min, 40% B for 4 min, 44% B for 5 min, 60% B for 13 min, 99% B for 4 min, then decreasing to 0% B for the remaining 10 min. A total of 48 fractions were eluted and collected, then concatenated down to 24 using the following lineup: 1 and 25, 2 and 26, 3 and 27, 4 and 28, and so on. Fractions were dried in a vacuum concentrator and vialed in 100 µl of 93% Buffer A (0.1% formic acid in water) and 7% Buffer B (0.1% formic acid in acetonitrile).

Mass spectrometry and raw data processing

TMT-labeled peptide fractions were resuspended in 2% acetonitrile/0.1% formic acid and ∼1 µg was loaded onto a heated PepMap RSLC C18 2 µm, 100 Å, 75 µm × 50 cm column (Thermo

bioRxiv preprint doi: https://doi.org/10.64898/2026.04.20.719668; this version posted April 22, 2026. The copyright holder for this preprint (which was not certified by peer review) is the author/funder, who has granted bioRxiv a license to display the preprint in perpetuity. It is made available under aCC-BY 4.0 International license.

Fisher, Cat. No. ES903) and eluted over a 180 min gradient. Sample eluate was electrosprayed (2,000 V) into a Thermo Scientific Orbitrap Eclipse mass spectrometer for analysis. MS1 spectra were acquired at a resolving power of 120,000. MS2 spectra were acquired in the ion trap with CID (35%) in centroid mode. Real-time search (max search time = 34 s; max missed cleavages = 1; Xcorr = 1; dCn = 0.1; ppm = 5) was used to select ions for synchronous precursor selection for MS3. MS3 spectra were acquired in the Orbitrap with HCD (60%) with an isolation window = 0.7 m/z and a resolving power of 60,000, and a max injection time of 400 ms. 4 µl (out of 20) of the TMT-labeled phosphopeptide enrichments were loaded onto a heated PepMap RSLC C18 2 µm, 100 Å, 75 µm × 50 cm column and eluted over a 180 min gradient: 1 min 2% B, 5 min 5% B, 160 min 25% B, 180 min 35% B. Sample eluate was electrosprayed (2,000 V) into a Thermo Scientific Orbitrap Eclipse mass spectrometer for analysis. MS1 spectra were acquired at a resolving power of 120,000. MS2 spectra were acquired in the Orbitrap with HCD (38%) in centroid mode with an isolation window = 0.4 m/z, a resolving power of 60,000, and a max injection time of 350 ms.

Raw MS files were processed in Proteome Discoverer version 2.4 (Thermo Fisher). MS spectra were searched against a custom FASTA database (see below). The SEQUEST search engine was used (enzyme = trypsin, max. missed cleavage = 3, min. peptide length = 6, precursor tolerance = 10 ppm). Static modifications included carbamidomethyl (C, +57.021 Da), and TMT labeling (N-term and K, +304.207 Da for TMTpro16). Dynamic modifications included oxidation (M, +15.995 Da), phosphorylation (S, T, Y, +79.966 Da, only for the phosphopeptide dataset), acetylation (N-term, +42.011 Da), Met-loss (N-term, −131.040 Da), and Met-loss + acetyl (N-term, −89.030 Da). Peptide spectral matches (PSMs) were filtered by the Percolator node (max Delta Cn = 0.05, target FDR (strict) = 0.01, and target FDR (relaxed) = 0.05). Proteins were identified with a minimum of 1 unique peptide and protein-level combined q-values < 0.05. Reporter ion quantification was based on intensity values with the following settings: integration tolerance = 20 ppm, method = most confident centroid, co-isolation threshold = 70, and SPS mass matches = 65.

Construction of brain region and cohort-specific FASTA file.

The RNAseq database was queried for transcripts expressed at levels of ≥ 3 counts-per-million in ≥ 75% of the 103 samples, which yielded 23,185 transcripts meeting this criteria. These sequences were then transformed into a cohort- and brain area-specific protein sequence database using a 3-frame translation. We then compared searches with the custom FASTA to standard databases (e.g., Swiss-Prot) and supplemented our list with the single canonical “protein” sequence from any gene not represented in the RNAseq dataset.

D Analysis of Isoform/Transcript Abundances

For the peptide dataset, we generated peptide measurements from samples of the anterior cingulate cortex (ACC) from brains preserved by the University of Pittsburgh brainbank. A tissue sample was taken from each of 56 individuals diagnosed with schizophrenia (case) and 56 unaffected individuals whose samples acted as controls. There were 46 matched case-control pairs (SEX, AGE, and PMI), which were always in the same plate and plex, and 10 cases and 10 controls that were evaluated as pairs, and they were similar in age and PMI, but not necessarily for sex. The samples were distributed over 8 plates; in addition to the 112 samples, 20 pooled samples (pools created from equal parts of all samples) were evaluated. Of the original 252,412 identified peptides, some had no measured abundance (46,214), and they were removed; likewise, peptides that could not be mapped to a protein (531) or peptides that had no detected abundance for at least half the samples and pools were removed (119,608). Remaining were 86,059 peptides for further analysis.

To impute missing values, starting values were obtained through the R package softImpute (*54*)

bioRxiv preprint doi: https://doi.org/10.64898/2026.04.20.719668; this version posted April 22, 2026. The copyright holder for this preprint (which was not certified by peer review) is the author/funder, who has granted bioRxiv a license to display the preprint in perpetuity. It is made available under aCC-BY 4.0 International license.

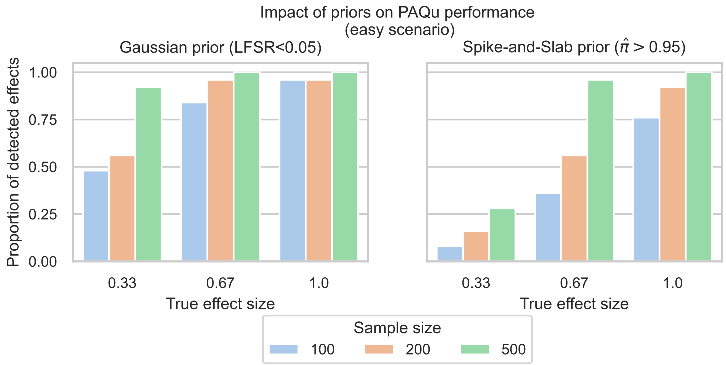

Figure 6: Simulation results, easy scenario, Gaussian vs spike-and-slab.

using the pooled samples as a reference. After this, a variational autoencoder (scVAEIT) (*55*) was applied only to the data from samples and thereby the final imputed values were obtained. Covariates plex, diagnosis, sex, ancestry, age, pmi, and pH (adjusted for the effect of DX, i.e., residual pH) were included as predictor variables in VAEIT's imputation process.

The imputed abundances for samples were normalized using sample lane normalization and then $\log_2$-transformed. Abundances were fit to a model with both biological (diagnosis, age, and postmortem interval) and technical predictors (plex) using ordinary least squares. The original abundance data were then adjusted for the effect of plex and pmi. By this process, we generated adjusted values for both transcripts and isoforms that still included effects for diagnosis and age. The resulting residuals were then used in subsequent analyses.

Transcript expression data were produced by the CommonMind Consortium (*25*) and were downloaded from
https://www.synapse.org, specifically syn29442530, accessed on February 4, 2025. Expression data were available from 473 samples. Subjects from whom samples were drawn were either diagnosed with schizophrenia or were unaffected (controls). These samples had count data for 207,749 transcripts. The observed expression counts were normalized using calcNormFactors from R package edgeR and subsequently counts per million (cpm) were calculated using cpm from the same R package. After this step, the data were reduced to 53,918 transcripts with at least 50% of the samples having cpm > 1. These expression data were then $\log_2$-transformed for further processing. Following (*56*), adjusted expression was calculated by fitting a model with both biological (diagnosis, sex, age, and RIN) and technical variables (intronic rate, interegenic rate, ribosomal RNA rate, and institution) using ordinary least squares. The original data expresssion data were then adjusted for the effect of the technical covariates, as well as sex and RIN. After obtaining the adjusted transcript expression, the data were limited to samples matching those from which peptide measurements were taken.

Across the two datasets, 103 samples were in common: the protein abundance dataset consisted of 86,059 peptides; the expression dataset consisted of 53,918 transcripts. After selecting peptides that could be mapped to transcripts, and vice versa, there were 9169 transcripts and 79,040 peptides remaining.

bioRxiv preprint doi: https://doi.org/10.64898/2026.04.20.719668; this version posted April 22, 2026. The copyright holder for this preprint (which was not certified by peer review) is the author/funder, who has granted bioRxiv a license to display the preprint in perpetuity. It is made available under aCC-BY 4.0 International license.

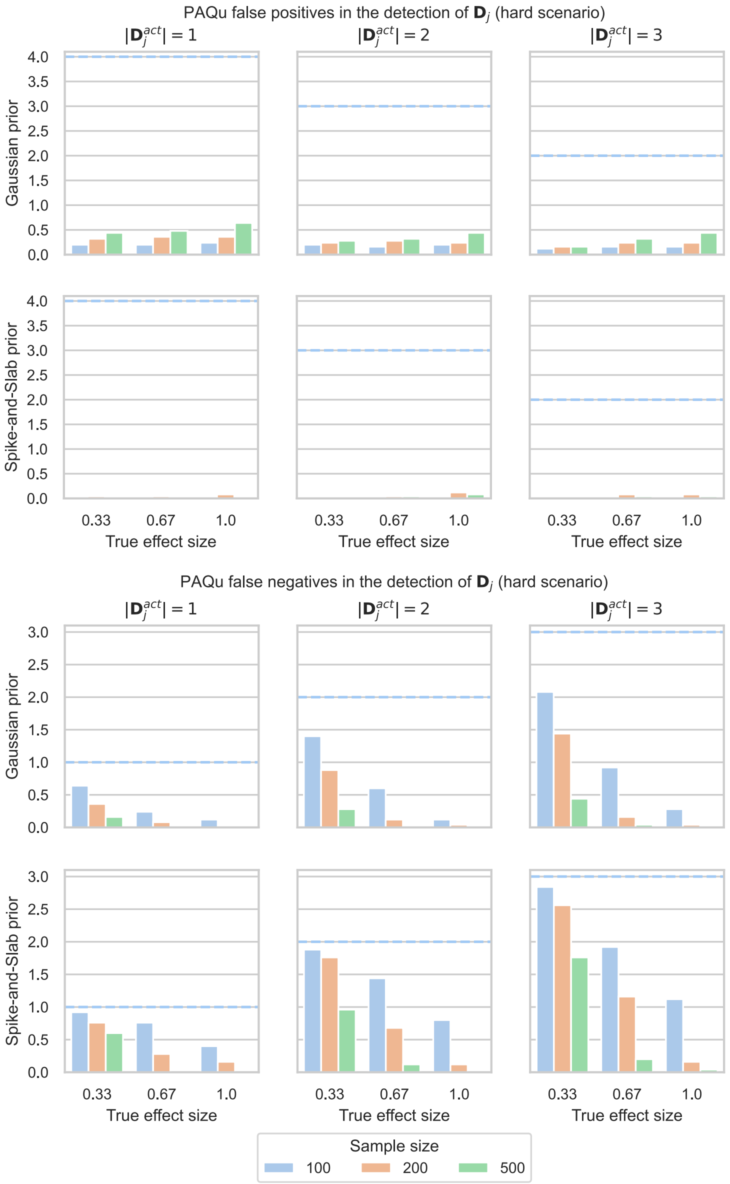

Figure 7: Simulation results, hard scenario, Gaussian vs spike-and-slab.

bioRxiv preprint doi: https://doi.org/10.64898/2026.04.20.719668; this version posted April 22, 2026. The copyright holder for this preprint (which was not certified by peer review) is the author/funder, who has granted bioRxiv a license to display the preprint in perpetuity. It is made available under aCC-BY 4.0 International license.

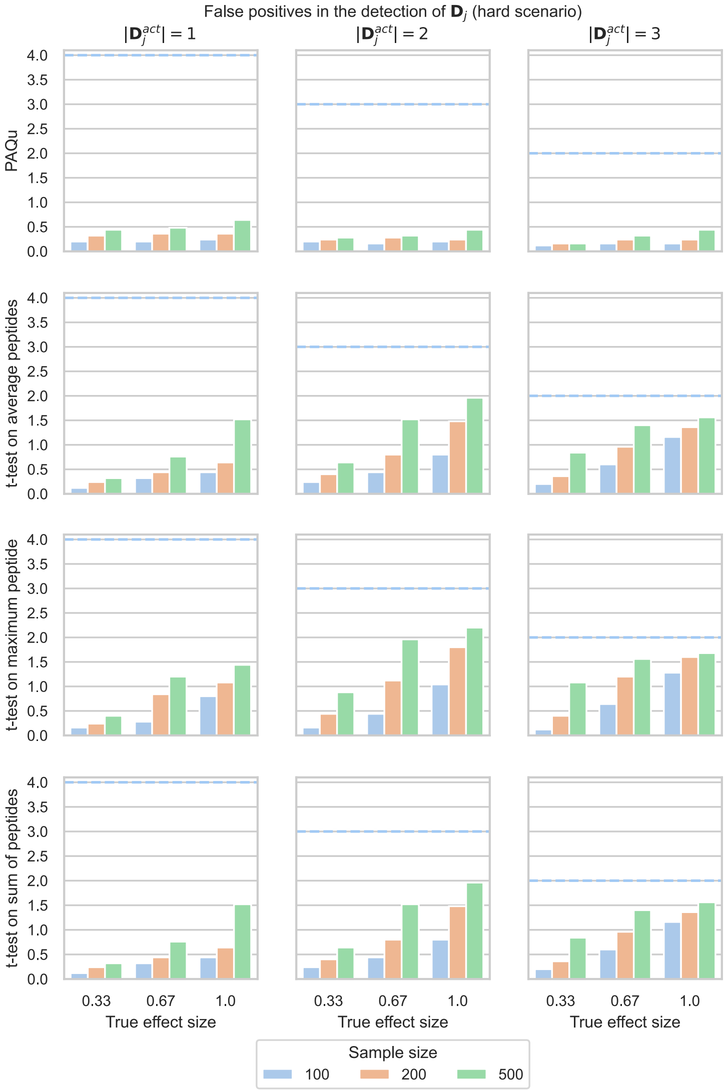

Figure 8: Simulation results, hard scenario, comparison. Blue dotted lines represent the maximum value the metric can take in any specific scenario.

bioRxiv preprint doi: https://doi.org/10.64898/2026.04.20.719668; this version posted April 22, 2026. The copyright holder for this preprint (which was not certified by peer review) is the author/funder, who has granted bioRxiv a license to display the preprint in perpetuity. It is made available under aCC-BY 4.0 International license.

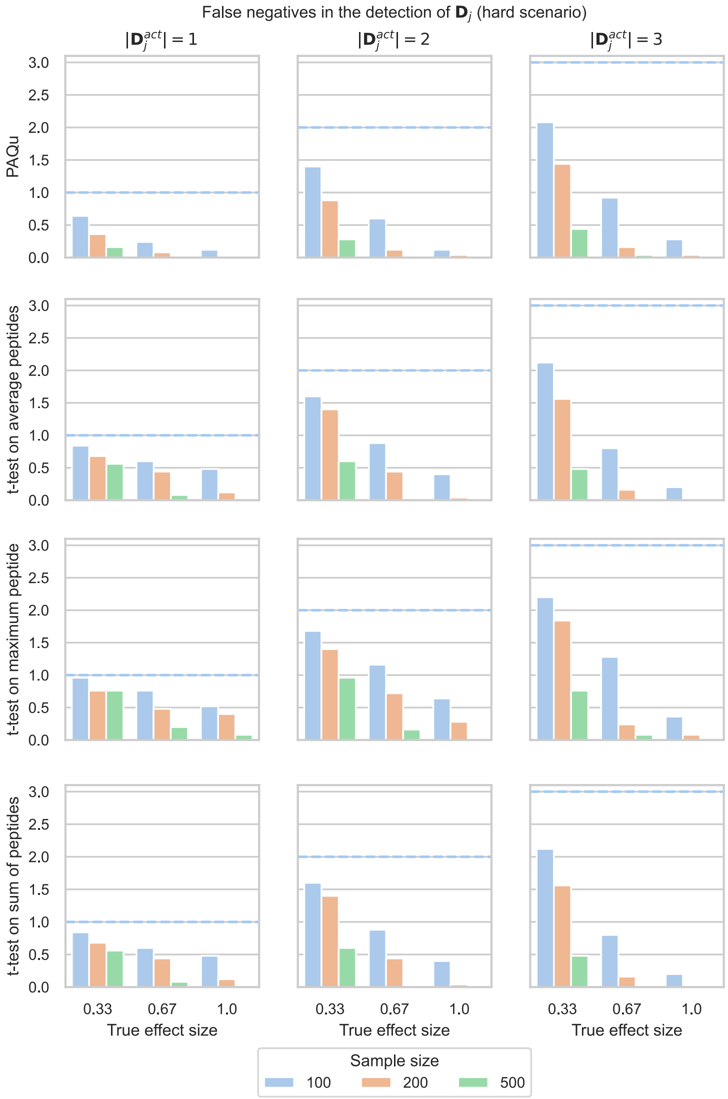

Figure 9: Simulation results, hard scenario, comparison. Blue dotted lines represent the maximum value the metric can take in any specific scenario.

bioRxiv preprint doi: https://doi.org/10.64898/2026.04.20.719668; this version posted April 22, 2026. The copyright holder for this preprint (which was not certified by peer review) is the author/funder, who has granted bioRxiv a license to display the preprint in perpetuity. It is made available under aCC-BY 4.0 International license.

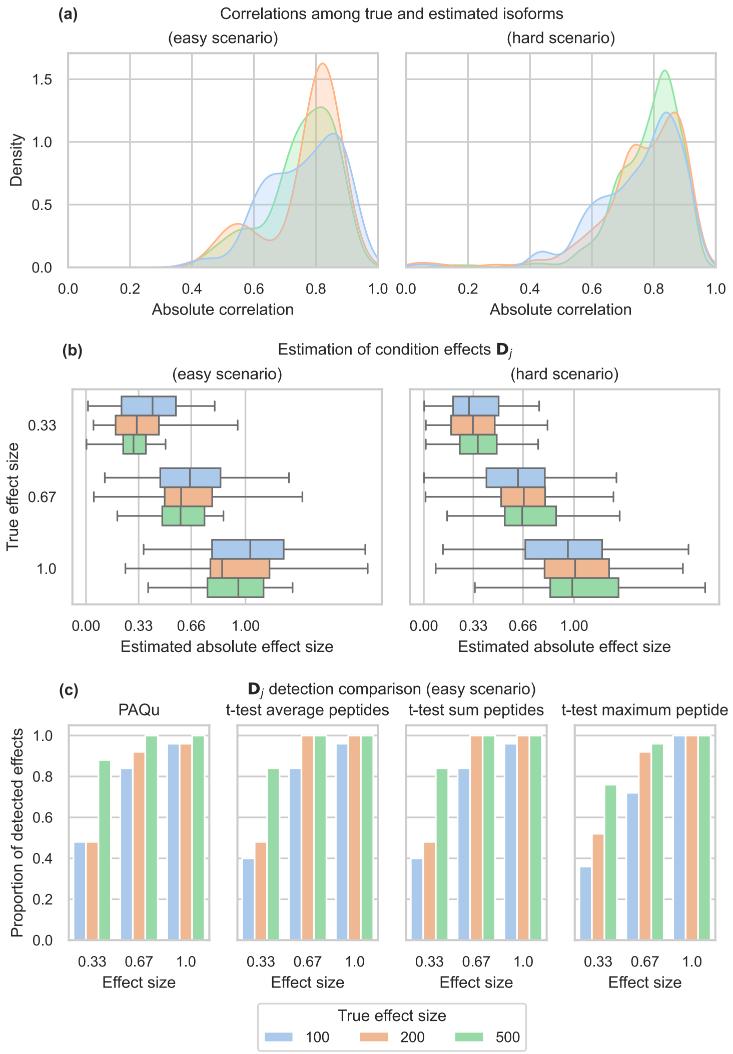

Figure 10: Simulation results, $\mathbf{W} = 0$.

bioRxiv preprint doi: https://doi.org/10.64898/2026.04.20.719668; this version posted April 22, 2026. The copyright holder for this preprint (which was not certified by peer review) is the author/funder, who has granted bioRxiv a license to display the preprint in perpetuity. It is made available under aCC-BY 4.0 International license.

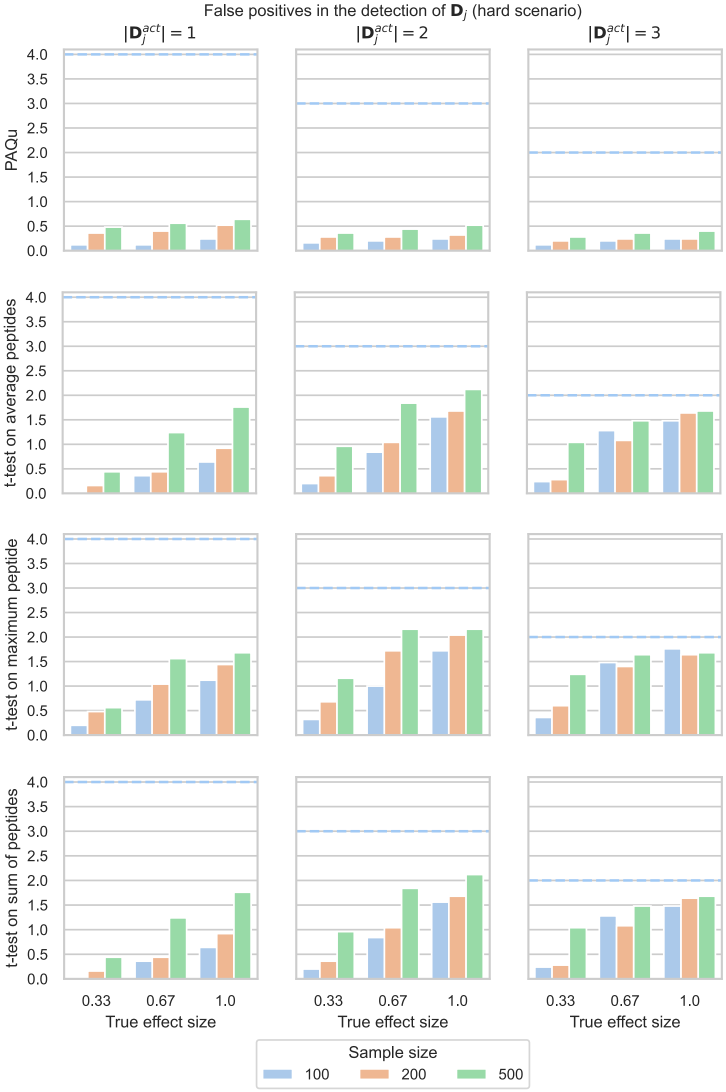

Figure 11: Simulation results, hard scenario, false positives, $\mathbf{W} = 0$.

bioRxiv preprint doi: https://doi.org/10.64898/2026.04.20.719668; this version posted April 22, 2026. The copyright holder for this preprint (which was not certified by peer review) is the author/funder, who has granted bioRxiv a license to display the preprint in perpetuity. It is made available under aCC-BY 4.0 International license.

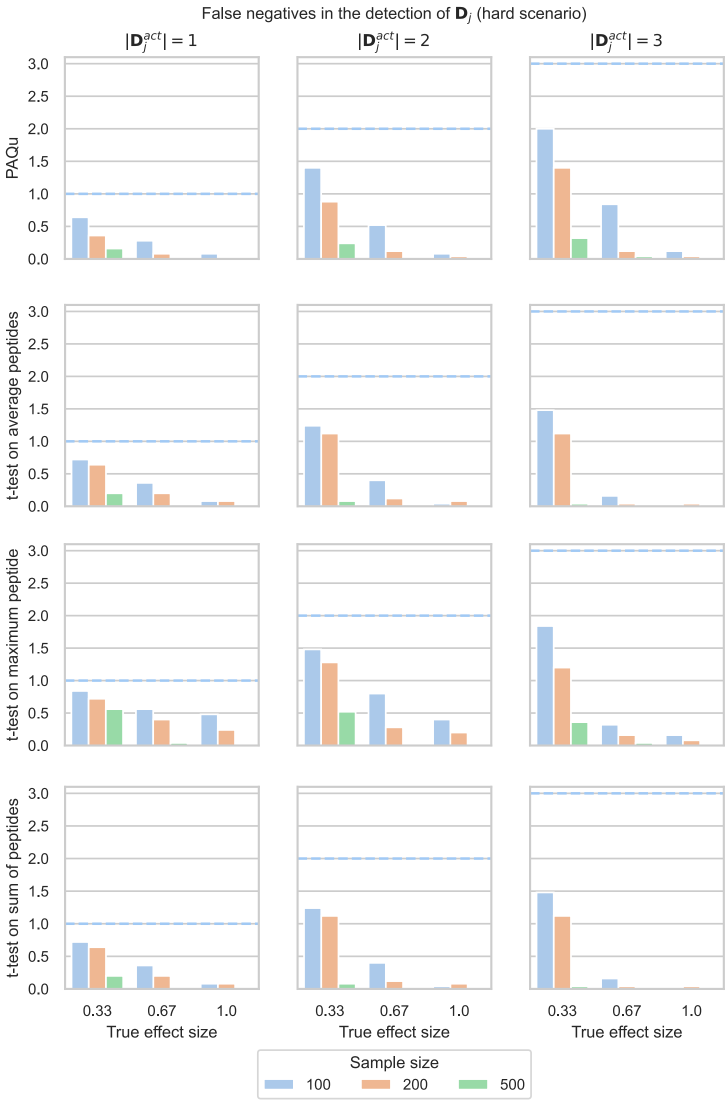

Figure 12: Simulation results, hard scenario, false negatives, $\mathbf{W} = 0$.

bioRxiv preprint doi: https://doi.org/10.64898/2026.04.20.719668; this version posted April 22, 2026. The copyright holder for this preprint (which was not certified by peer review) is the author/funder, who has granted bioRxiv a license to display the preprint in perpetuity. It is made available under aCC-BY 4.0 International license.

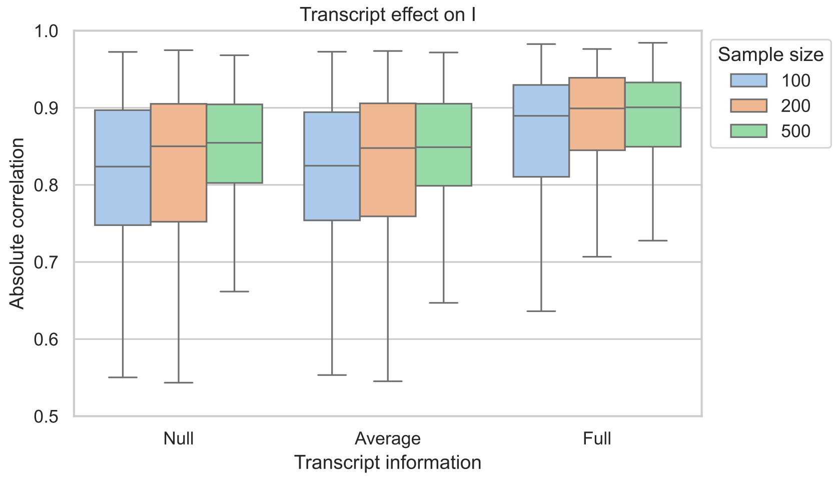

Figure 13: Effect on isoform abundance estimation of different availability of transcript information. $|\mathbf{D}^{act}| = 3$, $\mathbf{D}_j = 0.33$ for $j = 1, 2, 3$. Gaussian prior.

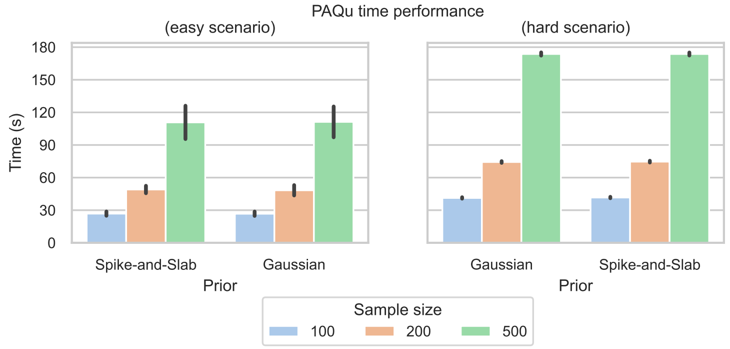

Figure 14: Simulation results, time.

bioRxiv preprint doi: https://doi.org/10.64898/2026.04.20.719668; this version posted April 22, 2026. The copyright holder for this preprint (which was not certified by peer review) is the author/funder, who has granted bioRxiv a license to display the preprint in perpetuity. It is made available under aCC-BY 4.0 International license.

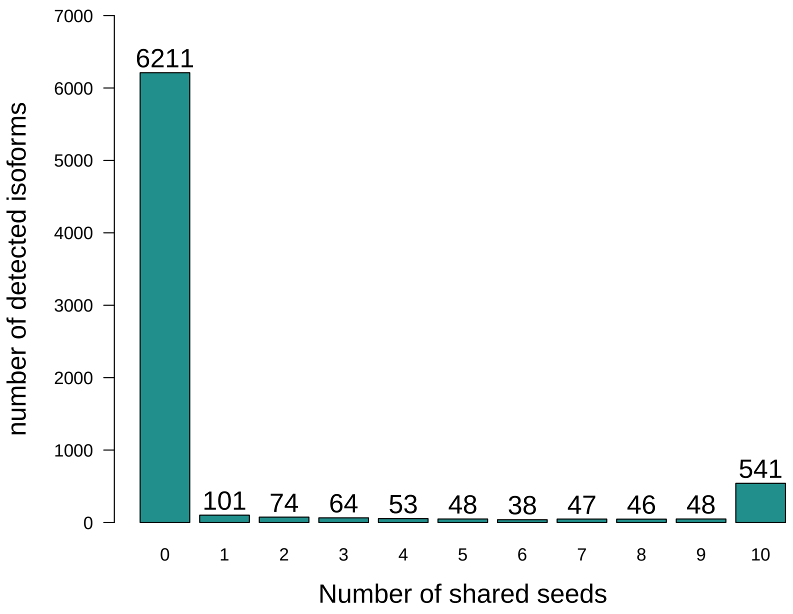

Figure 15: Isoform detection frequency as a function of the initial configuration seed

bioRxiv preprint doi: https://doi.org/10.64898/2026.04.20.719668; this version posted April 22, 2026. The copyright holder for this preprint (which was not certified by peer review) is the author/funder, who has granted bioRxiv a license to display the preprint in perpetuity. It is made available under aCC-BY 4.0 International license.

Table 1: Results from linear model analysis on three groups: both high and null correlations, only null correlations ($|r| < 0.05$), and high correlations ($r > 0.3$).

High and null correlation groups

Predictors[1]	Estimate	Std. Error	t value	P-value
intercept	0.184	0.008	23.574	0.000
diagnosis, isoform	0.225	0.080	2.808	0.005
age, isoform	-0.135	1.549	-0.087	0.931
diagnosis, transcript	0.035	0.049	0.716	0.474
age, transcript	4.831	1.325	3.646	0.000
genetic variation	0.370	0.126	2.938	0.003

Null correlation group

Predictors[1]	Estimate	Std. Error	t value	P-value
intercept	0.003	0.002	2.066	0.039
diagnosis, isoform	0.030	0.021	1.411	0.159
age, isoform	-0.450	0.418	-1.077	0.282
diagnosis, transcript	-0.001	0.011	-0.101	0.920
age,transcript	-0.437	0.277	-1.576	0.116
genetic variation	-0.012	0.034	-0.362	0.717

High correlation group[2]

Predictors[1]	Estimate	Std. Error	t value	P-value
intercept	0.388	0.005	76.082	0.000
diagnosis, isoform	0.246	0.045	5.457	0.000
age, isoform	-2.936	0.940	-3.125	0.002
diagnosis, transcript	-0.058	0.030	-1.917	0.056
age, transcript	2.220	0.898	2.471	0.014
genetic variation	0.103	0.066	1.559	0.120

[1]All predictors are estimated effects from analysis of the specific data type.
[2] How correlations of transcripts/isoform abundances arise: Following our notation in the manuscript, let T_{ijk} represent expression of subject i for transcript j and treatment k, which we could model as a function of mean μ_j, binary treatment difference δ_{jk} ($k = 0$ indicates controls, $k = 1$ othewise) and random error ϵ_{ijk}: $T_{ijk} = \mu_j + \delta_{jk} + \epsilon_{ijk}$. Let I_{ijk} be the corresponding isoform abundance, modeled in the same way: $I_{ijk} = \tau_j + \beta_{jk} + \eta_{ijk}$. We are interested in the correlation $\hat{r}_j$, the estimated correlation over i of T_{ijk} and I_{ijk}. In this setting, there are two sources of variation, measurement error for each omics and the difference between treatments. For our data, treatment would be affection status and effect the difference between their means for transcript (δ_{jk}) or isoform (β_{jk}). Measurement errors for transcript and isoform are independent, so only the case-control differences induce transcript/isoform correlation. Suppose we standardize T_{jk} and I_{jk} to have mean zero and variance 1, let $\delta_{jk} = \beta_{jk} = \{.25, .5, 1, or\ 1.5\}$ standard deviations (SD) when $k = 1$. The induced correlation is 0.014, 0.053, 0.2, and 0.36, respectively. For most empirical studies, however, an effect size of 0.25 SD is large. For instance, when (25) contrasted gene expression in the dorsolateral prefrontal cortex of 258 subjects with schizophrenia to 279 control samples, only 4.22% of effect sizes from 16,423 genes exceeded 0.25 SD and only one exceeded 0.5 SD. Thus, based on mean differences alone, we expect low correlations, generally $\hat{r} < 0.07$, and if there were no biological differences determining variation among subjects for transcript/isoform abundance, the correlation is expected to be zero.

bioRxiv preprint doi: https://doi.org/10.64898/2026.04.20.719668; this version posted April 22, 2026. The copyright holder for this preprint (which was not certified by peer review) is the author/funder, who has granted bioRxiv a license to display the preprint in perpetuity. It is made available under aCC-BY 4.0 International license.

Table 2: GO Cellular Component and Biological Process Analysis

GO cellular component	Genes in set	Observed genes	Expected	Fold Enrich-ment	FDR	GO Type
actin-based cell projection (GO:0098858)	97	19	6.95	2.74	0.00371	CC
adherens junction (GO:0005912)	112	18	8.02	2.24	0.0463	CC
apical part of cell (GO:0045177)	177	25	12.67	1.97	0.0398	CC
astrocyte projection (GO:0097449)	16	6	1.15	5.24	0.0301	CC
axon (GO:0030424)	447	52	32.01	1.62	0.0268	CC
blood microparticle (GO:0072562)	35	13	2.51	5.19	0.0000997	CC
brush border (GO:0005903)	44	12	3.15	3.81	0.00355	CC
cell junction (GO:0030054)	1467	146	105.04	1.39	0.000596	CC
cell periphery (GO:0071944)	1969	195	140.98	1.38	0.00000713	CC
cell surface (GO:0009986)	265	40	18.97	2.11	0.000619	CC
cluster of actin-based cell projections (GO:0098862)	61	17	4.37	3.89	0.000161	CC
external encapsulating structure (GO:0030312)	115	27	8.23	3.28	0.00000849	CC
extracellular matrix (GO:0031012)	115	27	8.23	3.28	0.00000679	CC
extracellular region (GO:0005576)	1436	141	102.82	1.37	0.00127	CC
extracellular space (GO:0005615)	1272	128	91.08	1.41	0.00132	CC
intermediate filament (GO:0005882)	29	8	2.08	3.85	0.0354	CC
intermediate filament cytoskeleton (GO:0045111)	34	9	2.43	3.70	0.0272	CC
neuron projection (GO:0043005)	785	81	56.21	1.44	0.0272	CC
plasma membrane (GO:0005886)	1842	172	131.89	1.30	0.00185	CC
presynapse (GO:0098793)	474	53	33.94	1.56	0.0357	CC
synapse (GO:0045202)	1164	110	83.34	1.32	0.0459	CC
synaptobrevin 2-SNAP-25-syntaxin-1a complex (GO:0070044)	7	4	0.5	7.98	0.0361	CC
anterograde trans-synaptic signaling (GO:0098916)	226	34	16.18	2.1	0.0141	BP
carbon dioxide transport (GO:0015670)	4	4	0.29	13.97	0.0137	BP
cell adhesion (GO:0007155)	362	51	25.92	1.97	0.00523	BP
cell-cell signaling (GO:0007267)	343	48	24.56	1.95	0.00389	BP
chemical synaptic transmission (GO:0007268)	226	34	16.18	2.1	0.0147	BP
kidney epithelium development (GO:0072073)	34	10	2.43	4.11	0.0313	BP
modulation of chemical synaptic transmission (GO:0050804)	327	47	23.41	2.01	0.00389	BP
multicellular organismal process (GO:0032501)	2042	185	146.21	1.27	0.0205	BP
nervous system process (GO:0050877)	364	50	26.06	1.92	0.00396	BP
one-carbon compound transport (GO:0019755)	5	4	0.36	11.17	0.0412	BP
regulated exocytosis (GO:0045055)	82	18	5.87	3.07	0.00848	BP
regulation of ERK1 and ERK2 cascade (GO:0070372)	94	20	6.73	2.97	0.006	BP
regulation of synaptic transmission glutamatergic (GO:0051966)	41	11	2.94	3.75	0.0342	BP
regulation of trans-synaptic signaling (GO:0099177)	328	47	23.49	2	0.00364	BP
synaptic signaling (GO:0099536)	267	39	19.12	2.04	0.00827	BP
synaptic vesicle docking (GO:0016081)	13	6	0.93	6.45	0.0459	BP
system process (GO:0003008)	642	75	45.97	1.63	0.00692	BP
trans-synaptic signaling (GO:0099537)	245	37	17.54	2.11	0.00694	BP
